# The Epstein-Barr Virus Glycoprotein gp150 Forms an Immune-Evasive Glycan Shield at the Surface of Infected Cells

**DOI:** 10.1371/journal.ppat.1005550

**Published:** 2016-04-14

**Authors:** Anna M. Gram, Timo Oosenbrug, Marthe F. S. Lindenbergh, Christian Büll, Anouskha Comvalius, Kathryn J. I. Dickson, Joop Wiegant, Hans Vrolijk, Robert Jan Lebbink, Ron Wolterbeek, Gosse J. Adema, Marieke Griffioen, Mirjam H. M. Heemskerk, David C. Tscharke, Lindsey M. Hutt-Fletcher, Emmanuel J. H. J. Wiertz, Rob C. Hoeben, Maaike E. Ressing

**Affiliations:** 1 Department of Molecular Cell Biology, Leiden University Medical Center, Leiden, The Netherlands; 2 Department of Medical Microbiology, University Medical Center Utrecht, Utrecht, The Netherlands; 3 Department of Tumor Immunology, Radboud Institute for Molecular Life Sciences, Nijmegen, The Netherlands; 4 John Curtin School of Medical Research, Australian National University, Canberra, Australia; 5 Department of Medical Statistics & Bioinformatics, Leiden University Medical Center, Leiden, The Netherlands; 6 Department of Hematology, Leiden University Medical Center, Leiden, The Netherlands; 7 Department of Microbiology and Immunology, Louisiana State University Health Sciences Center, Shreveport, Louisiana, United States of America; Oregon Health & Science University, UNITED STATES

## Abstract

Cell-mediated immunity plays a key role in host control of viral infection. This is exemplified by life-threatening reactivations of e.g. herpesviruses in individuals with impaired T-cell and/or iNKT cell responses. To allow lifelong persistence and virus production in the face of primed immunity, herpesviruses exploit immune evasion strategies. These include a reduction in viral antigen expression during latency and a number of escape mechanisms that target antigen presentation pathways. Given the plethora of foreign antigens expressed in virus-producing cells, herpesviruses are conceivably most vulnerable to elimination by cell-mediated immunity during the replicative phase of infection. Here, we show that a prototypic herpesvirus, Epstein-Barr virus (EBV), encodes a novel, broadly acting immunoevasin, gp150, that is expressed during the late phase of viral replication. In particular, EBV gp150 inhibits antigen presentation by HLA class I, HLA class II, and the non-classical, lipid-presenting CD1d molecules. The mechanism of gp150-mediated T-cell escape does not depend on degradation of the antigen-presenting molecules nor does it require gp150’s cytoplasmic tail. Through its abundant glycosylation, gp150 creates a shield that impedes surface presentation of antigen. This is an unprecedented immune evasion mechanism for herpesviruses. In view of its likely broader target range, gp150 could additionally have an impact beyond escape of T cell activation. Importantly, B cells infected with a gp150-null mutant EBV displayed rescued levels of surface antigen presentation by HLA class I, HLA class II, and CD1d, supporting an important role for iNKT cells next to classical T cells in fighting EBV infection. At the same time, our results indicate that EBV gp150 prolongs the timespan for producing viral offspring at the most vulnerable stage of the viral life cycle.

## Author Summary

The human herpesvirus Epstein-Barr virus (EBV) is an important human pathogen involved in infectious mononucleosis and several malignant tumors, including lymphomas in the immunosuppressed. Upon primary infection, a balance between virus and host is established, to which EBV’s capacity to dodge T cell-mediated attack contributes. Here we identify the late protein EBV gp150 as a novel immunoevasin, frustrating antigen presentation by HLA class I, class II, and CD1d molecules. EBV gp150’s many sialoglycans create a shield impeding surface detection of presented antigen. Interestingly, exploiting glycan shielding as a mechanism to mask surface exposed proteins on infected cells could permit EBV to additionally modulate other aspects of host antiviral defense. B cells producing wild-type EBV escaped immune recognition more efficiently than those infected with a gp150-null virus, pointing towards a role for gp150 in natural infection. Our results reveal a novel, broadly active strategy by which a herpesvirus glycoprotein, EBV gp150, blocks antigen presentation to T cells through glycan shielding, a new paradigm in herpesvirus immune evasion.

## Introduction

Viruses are exceptionally well equipped to adjust processes in infected host cells to support their own replication and survival. Especially in persistent infections, they must withstand many layers of anti-viral activities exerted by the host immune system. Cell-mediated immunity, in particular that mediated by antigen (Ag)-specific T cells, is essential for elimination of virus-infected cells, reducing viral replication, and stimulating other immune effector functions.

CD8^+^ and CD4^+^ T cells are activated by peptide Ags presented at the cell surface in the context of HLA class I and class II (HLA I and II) molecules, respectively. In contrast, invariant natural killer T (iNKT) cells, a subset of specialized T cells characterized by a semi-invariant T cell receptor (TCR) in combination with NK cell receptors, recognize lipid Ags presented by CD1d, a non-classical, non-polymorphic HLA molecule. Upon activation, iNKT cells secrete a vast array of polarizing cytokines and they can also directly exert cytotoxicity [1]. In addition to the anti-viral cytotoxic and helper T cells, a role for iNKT cells in providing protection against viral infection has more recently been appreciated [2].

Herpesviruses are widespread viruses that establish lifelong persistent infections in their host, even in the face of virus-specific immunity [3]. Within their large (125–230 kb) DNA genomes, herpesviruses encode functions essential for viral replication, yet many additional gene products are non-essential for propagation *in vitro*. Evidence is accumulating that the latter are required for establishment of the delicate balance between viral replication and host responses *in vivo*. Among these gene products are immune evasion molecules that frustrate the activation of cell-mediated immunity by thwarting Ag presentation. The fact that such gene products have been independently acquired and maintained by multiple members of the herpesvirus family throughout millions of years of coevolution with their host testifies to the importance of T cell activation in the control of herpesvirus infection [4].

In humans, the *in vivo* contribution of cell-mediated immunity to fighting viral infection is probably best demonstrated for Epstein-Barr virus (EBV). This γ-herpesvirus was the first human tumor virus discovered [5], being associated with malignancies such as Burkitt’s lymphoma and nasopharyngeal carcinoma. EBV is carried by more than 90% of adults worldwide, mostly asymptomatically. When primary EBV infection occurs during adolescence or adulthood, it presents in more than 50% of cases as self-limiting infectious mononucleosis [6], in which viral replication is eventually controlled by the EBV-specific immune response. This contrasts to patients with the primary immunodeficiency X-linked lymphoproliferative syndrome (XLP) who develop an often fatal mononucleosis when infected by EBV [6]. XLP patients have a genetic defect leading to nonfunctional T and NK cells and the absence of iNKT cells [7,8]. In addition, EBV can cause life-threatening lymphomas when cell-mediated immunity is insufficient, for instance in transplantation or AIDS patients. Reconstitution of EBV-specific T cell immunity by adoptive transfer provides a cure for post-transplantation lymphoproliferative disease (PTLD) [9]. PTLD and XLP thus occur in two distinct patient groups having in common that they lack sufficient cell-mediated immunity to control EBV infection.

Like other herpesviruses, EBV has acquired ingenious strategies to escape elimination by cell-mediated immunity of the immunocompetent infected host. Among these, EBV downregulates its Ag expression in the latent stage of infection and exploits (viral) immune evasion gene products, in particular during its replicative phase, to interfere with immune activation [10]. In the early lytic phase, Ag presentation by HLA I is reduced by the concerted action of (at least) three EBV proteins: BNLF2a blocks antigenic peptide supply [11], BILF1 diverts HLA I complexes away from the cell surface [12], and the shutoff protein BGLF5 acts as an RNase that arrests synthesis of, for instance, HLA I, II, and CD1d [10]. Silencing expression of these early EBV proteins in B cells supporting productive infection yielded only partially rescued surface display of the Ag-presenting molecules [13,14]. This led to the hypothesis that EBV encodes additional immune evasion molecules targeting CD1d, HLA I, and HLA II, but their identity has remained elusive. In this study, we took a global look at Ag presentation by EBV-producing B cells with a focus on the late phase of infection, when most Ags are expressed that could activate T and iNKT cell immunity.

We identified the heavily glycosylated EBV protein gp150, encoded by the *BDLF3* gene, as a new viral immune evasion molecule. *BDLF3* is unique to EBV and the closely related Rhesus lymphocryptovirus (LCV). EBV gp150 interferes with immune recognition of HLA I, II, and CD1d/ Ag complexes at the cell surface. We provide evidence that gp150 shields the recognition of these surface molecules in a glycan-dependent manner. This represents a novel immune evasion mechanism in herpesviruses.

## Results

### Identification of EBV *BDLF3*-encoded gp150 as a novel immune evasion molecule

**Fig 1:**
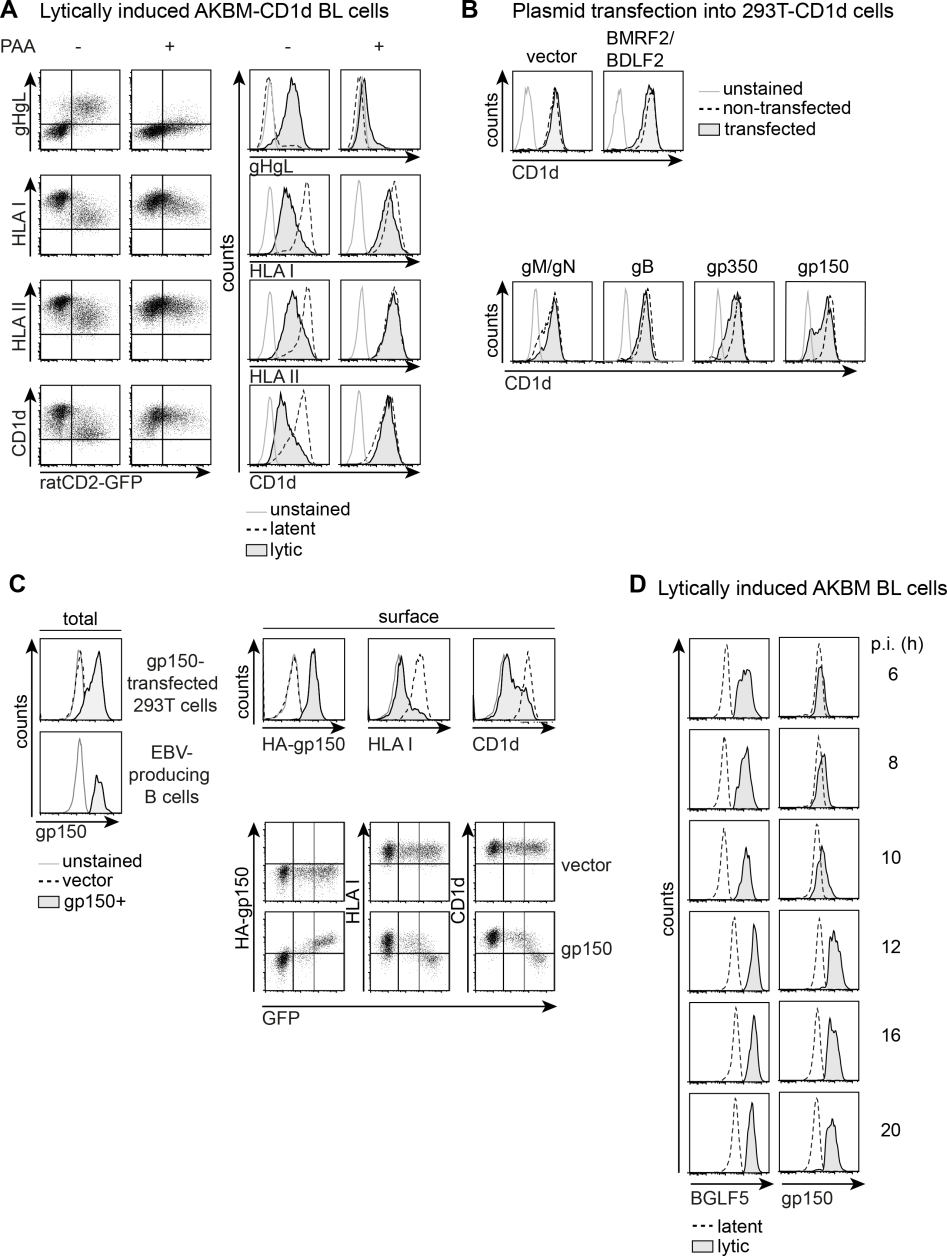
gp150 interferes with surface display of Ag-presenting molecules during productive EBV infection. A) EBV^+^ AKBM-CD1d BL cells were treated for 20 hours with anti-human IgG Ab to induce viral replication. EBV-producing cells were identified by induced expression of the lytic cycle reporter rat CD2-GFP. In the presence of PAA, productive infection is arrested before late protein expression. Surface levels of the EBV late complex gHgL and of the Ag-presenting molecules HLA I, II, and CD1d were determined by flow cytometry. Histograms depict overlays to allow comparison of latently (GFP^-^) and lytically (GFP^+^) infected B cells. B) 293T-CD1d cells were transfected with expression vectors encoding late EBV glycoproteins. Glycoproteins known to require a viral interaction partner were transfected together (BMRF2/BDLF2 and gM/gN). EBV protein expression was deduced from coexpression of GFP (BMRF2/BDLF2) or on the basis of a C-terminal tag (gM/gN, gB, gp350, gp150). Cell surface CD1d was stained prior to an intracellular staining for the tagged EBV proteins. Surface levels were compared between non-transfected and transfected cells. C) 293T-CD1d cells were transfected with N-terminally HA-tagged gp150 or an empty IRES-GFP vector control. Total gp150 expression was determined by intracellular staining and was compared to levels in EBV-producing B cells. The other histograms depict surface levels of HA (HA-gp150), HLA I, and CD1d staining for empty vector-transfected and gp150^+^GFP^high^ 293T-CD1d cells; GFP^high^ gating as indicated in the dot plots. D) Expression of gp150 was assessed at different time points during productive EBV infection of B cells; the early EBV antigen BGLF5 was taken along as a control (time points 6–10 hours and 12–20 hours are from different experiments).

To study alterations in surface display of Ag-presenting molecules in the course of productive EBV infection of B cells, we employed our previously described EBV^+^ AKBM Burkitt’s lymphoma (BL) system [15]. Following anti-human IgG treatment, cells supporting replication of EBV are identified in flow cytometry by expression of a lytic cycle reporter, rat CD2-GFP, and/or EBV lytic proteins, such as the gHgL complex (Fig 1A, upper row). Surface display of the Ag-presenting molecules HLA I, II, and the non-classical HLA molecule CD1d is markedly reduced on EBV-producing cells in comparison to latently infected control cells (Fig 1A, left panels), in line with our earlier reports [13,15]. Downregulation of surface HLA I complexes commenced around 8–10 hours post induction of the lytic cycle [15], preceding that of HLA II and CD1d. Surface levels of the latter were progressively reduced on EBV-producing cells with a delay of about 4–6 hours compared to HLA I (S1A Fig and [15]). To distinguish the contributions of late versus (immediate-)early EBV proteins to the downregulation of surface Ag-presenting molecules, phosphonoacetic acid (PAA) was used to arrest viral replication at the early phase. Upon anti-human IgG treatment of AKBM cells in the presence of PAA, late EBV protein expression of, for instance, gHgL complexes was inhibited, while the early rat CD2GFP reporter was still expressed (Fig 1A, upper row). At the surface of PAA-treated cells, CD1d and HLA II levels were mostly retained (Fig 1A, right panels), pointing towards involvement of late EBV protein(s) in T and iNKT cell immune evasion. HLA I downregulation was partly rescued upon PAA treatment indicating that, next to the early EBV proteins previously identified, also viral late proteins play a role in reducing the surface levels of HLA I (Fig 1A, right panels). In conclusion, one or several of the 30 late EBV proteins interfere with surface display of the CD1d, HLA I, and HLA II Ag-presenting molecules.

To identify the late EBV gene product(s) responsible for interference with Ag presentation observed during productive infection, we took the approach of expressing individual, or combinations of late viral genes in 293T-CD1d cells through plasmid transfection (Fig 1B and 1C) and/or in MJS-CD1d cells via lentiviral transduction (Figs 1D and S1). Screening of late EBV genes in 293T-CD1d cells revealed that most glycoproteins did not affect surface levels of CD1d (Fig 1B), HLA I, or the control protein transferrin receptor (TfR) to a major extent (S1B Fig). Also expression of EBV gB did not target CD1d, even though its homologue in herpes simplex virus type 1 (HSV-1) was found to act in concert with the viral kinase US3 to mediate intracellular retention of CD1d molecules observed during HSV-1 infection [16]. EBV gp350 caused some downregulation of CD1d, yet the strongest effect was observed for gp150, encoded by the *BDLF3* gene (Fig 1B). Expression of EBV gp150 at levels comparable to those observed in virus-producing B cells (Fig 1C, left panels) resulted in marked downregulation of both Ag-presenting molecules tested (upper panels), which was not evident in control transfected cells. The dot plots show an apparent threshold to gp150 levels resulting in loss of HLA I and CD1d detection. These combined data demonstrate that expression of the *BDLF3*-encoded EBV gp150 reduces the detection of HLA I and CD1d at the cell surface.

Finally, we determined whether the timing of gp150 expression during productive EBV infection of human B cells would match the observed downregulation of Ag-presenting molecules. To visualize total gp150 protein levels in the course of EBV reactivation in B cells, we used an antibody (Ab) directed against its intracellular cytoplasmic tail [19]. Whereas the early EBV protein BGLF5 was detectable from 4 hours post induction [15] onwards, no gp150 protein was found before 8–10 hours into lytic infection with clear detection from 12 hours onwards (Fig 1D), leaving enough time to influence HLA I, II, and CD1d expression at late times. These combined data point to gp150 interfering with surface detection of Ag-presenting molecules on EBV-producing B cells. Since no function had hitherto been ascribed to the EBV glycoprotein gp150, its role in immune evasion of Ag-presenting molecules was further investigated.

### EBV gp150 causes expression level-dependent interference with different cell surface molecules

To study gp150’s phenotype and mechanism of action in more detail, we expressed the EBV protein in MJS-CD1d cells via lentiviral transduction, together with GFP as a marker for successful transduction. This approach was validated with known EBV evasion molecules [11,17,18] that specifically reduced surface display of HLA I (BNLF2a and BILF1) or HLA II (gp42gHgL). At the same time, this experiment showed that the known EBV T cell immunoevasins did not interfere with CD1d expression (S2A Fig).

**Fig 2:**
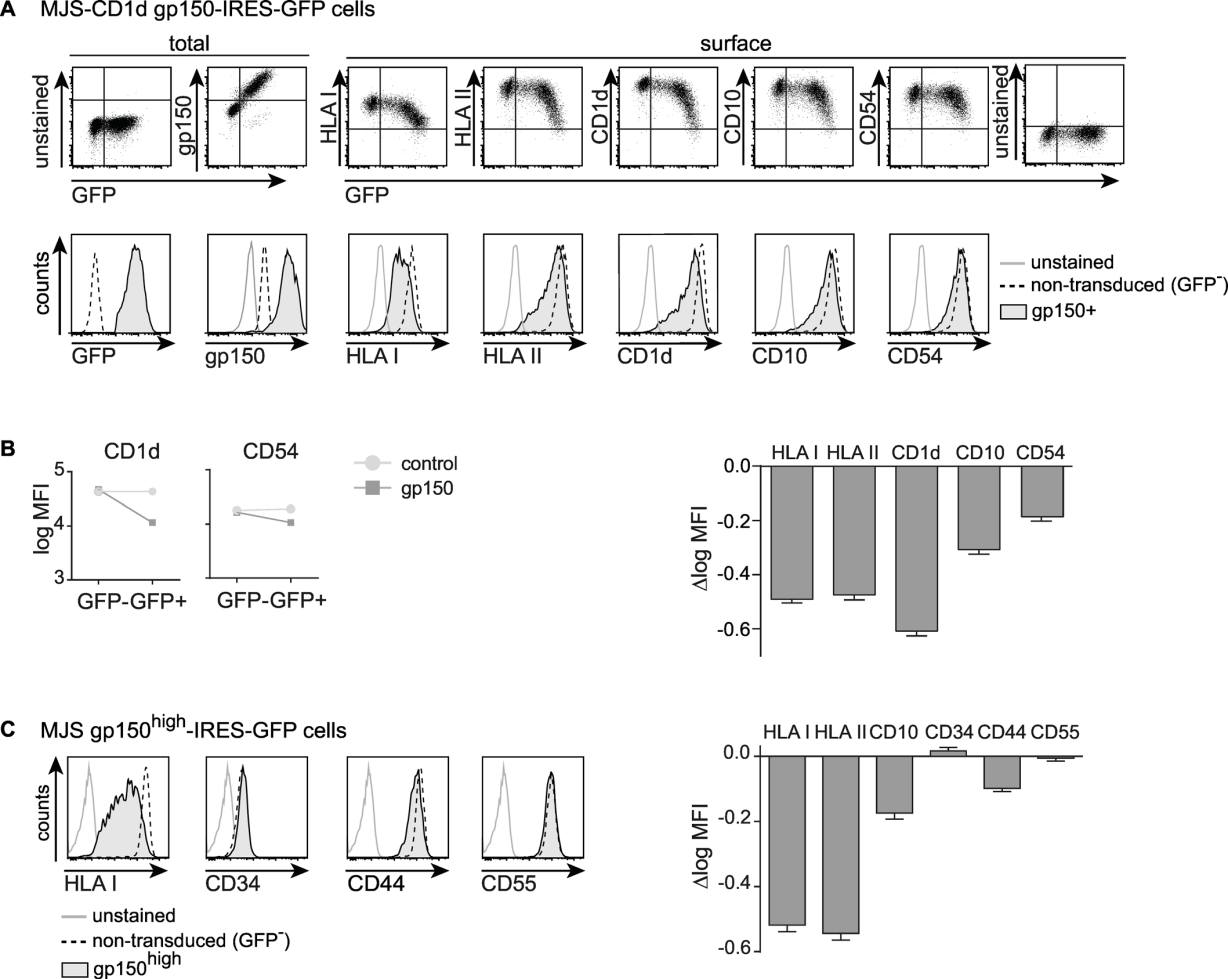
EBV gp150 is broadly acting, but displays a degree of specificity for antigen-presenting molecules. Flow cytometric analyses of MJS-CD1d cells (adherent fraction) three days post transduction with gp150-IRES-GFP lentiviruses. A) Total EBV gp150 levels were determined by intracellular staining of permeabilized cells. Surface levels of HLA I, II, CD1d, CD10, and CD54 were determined using Ab staining on non-permeabilized cells. B) Surface levels of CD1d and CD54 are depicted as log MFI values with 95% confidence intervals, for GFP^+^ versus GFP^-^ MJS-CD1d cells transduced with gp150 or a GFP control. The slopes of the connecting lines reflect the declines in fluorescence (Δlog MFI) and, thus, the downregulation of HLA I, II, CD1d, CD10, and CD54 induced by gp150 (for the GFP control, the Δlog MFI did not significantly differ from 0). C) Cell surface levels of HLA I, II, CD10, CD34, CD44, and CD54 were determined using surface Ab staining. EBV gp150-induced downregulation of these molecules was calculated as in B) and represented as Δlog MFI.

HLA I and CD1d downregulation from the GFP^+^ MJS-CD1d-gp150 cells (Figs 2A and S2B) indicated that this immune evasive phenotype was not limited to transient overexpression in 293T cells (Fig 1). Furthermore, gp150 downregulated surface levels of HLA II and also affected other molecules such as CD10, CD54 (Fig 2A), and TfR (S2B Fig). To allow easier comparison of gp150’s effects on various cell surface markers, we calculated the difference in logarithmically transformed mean fluorescence intensities (Δlog MFI) for each surface marker to serve as a measure for the extent of gp150-mediated downregulation, by subtracting the log MFI values for cells that were non- (GFP^-^) or successfully (GFP^+^) transduced with either gp150-IRES-GFP or a control GFP lentivirus (Fig 2B right panel; the left panels show examples of the log MFI for CD1d and CD54). This approach visualized that the antigen-presenting molecules HLA I, II and CD1d were more sensitive to EBV gp150 downregulation than the CD10 and CD54 molecules (Fig 2B).

To investigate if gp150’s effects reflect level-dependency, we transduced MJS-CD1d cells with a range of lentivirus amounts to achieve decreasing levels of gp150 expression. This resulted in a concomitant reduction in surface downregulation of the cellular proteins tested (S2C Fig). When using the higher dose of gp150 lentiviruses in the adherent MJS-CD1d cells, we observed that a proportion of cells detached from the culture dish by 3 days after transduction. This detachment did not only occur as a consequence of lentiviral transduction or of gross gp150-induced cytotoxicity (see S3A Fig). Loss of attachment of the gp150^high^ GFP^high^ cells coincided with a further reduced surface detection of HLA I, II, and CD1d (compare Figs 2A and S3B). Very high levels of gp150 even downregulated the cell surface molecules CD10 and CD54, which were only marginally affected in the adherent cell fraction (Figs 2A and S3B). These results—together with the apparent threshold for gp150-induced downregulation (dot plots in Figs 1C and 2A) and the dose-dependent effect of gp150 expression (S2C Fig)—demonstrate that higher levels of gp150 cause a stronger phenotype.

Thus, EBV gp150 acts in an expression level-dependent manner to downregulate multiple cell surface molecules, to different extents.

Finally, to obtain an impression of the target range of gp150, we looked for surface molecules on MJS-CD1d cells that were not downregulated by the EBV protein. We found that virtually equal levels of CD34, CD44, and CD55 were detected on cells that did or did not express gp150, even when gated on the GFP^high^ fractions of cells (Fig 2C). From these data we conclude that various cell surface molecules display differential sensitivity to EBV gp150, with CD34, CD44, and CD55 as examples of proteins that are resistant (Fig 2). Since gp150 has a strong effect on the Ag-presenting molecules HLA I, II, and CD1d, this EBV glycoprotein appears to be a broadly acting immune evasion molecule.

### EBV gp150-induced downregulation of Ag-presenting molecules impairs T cell activation

Next, we intended to determine the functional consequences of EBV gp150-induced immune evasion. Reduced surface HLA I, II, and CD1d levels were detected with Abs that are capable of blocking many TCR–HLA/Ag interactions (Figs 1 and 2), pointing towards functional relevance of gp150’s blocking activity.

**Fig 3:**
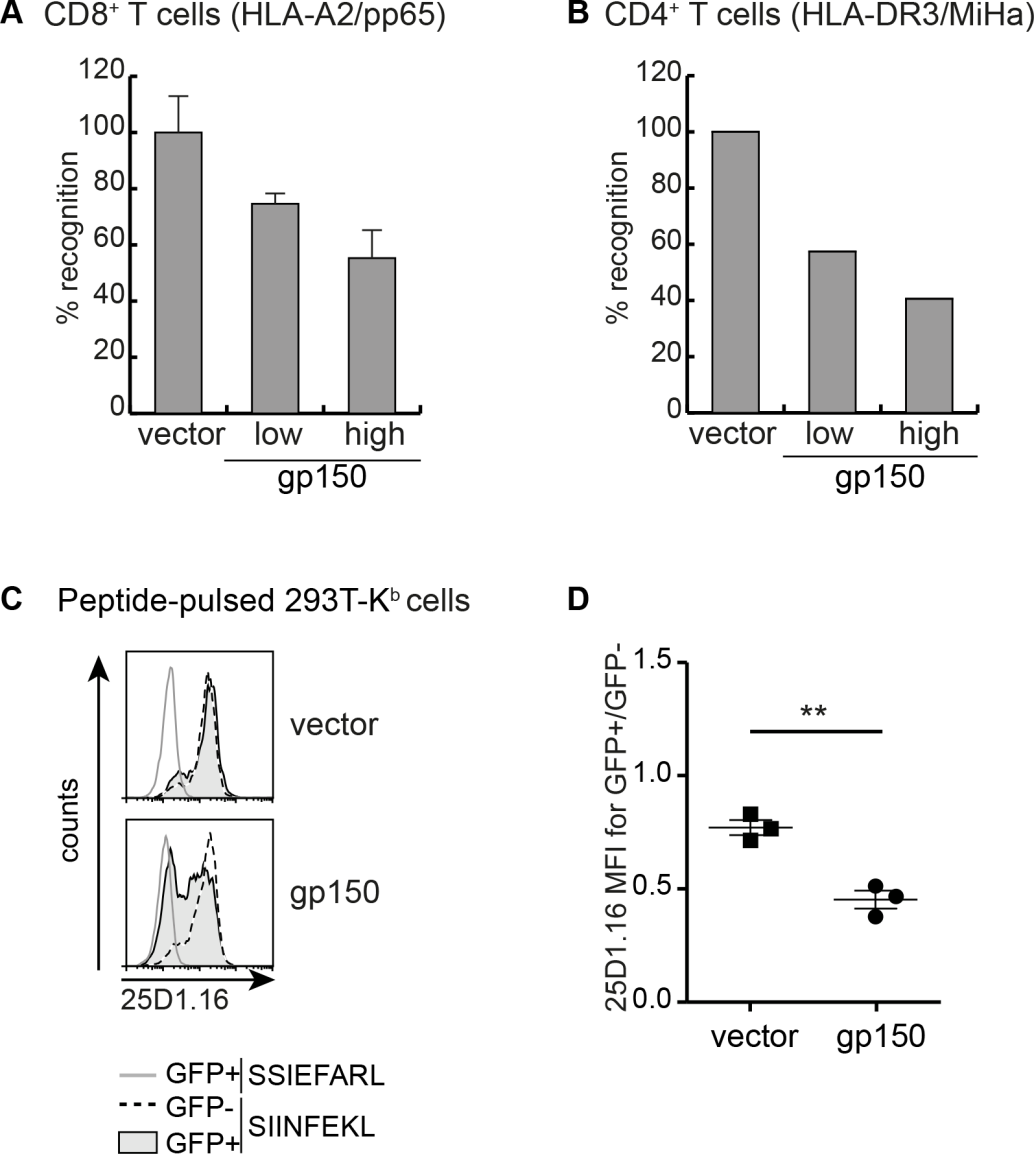
Cellular expression of EBV gp150 limits CD8^+^ and CD4^+^ T cell activation by disrupting Ag presentation. A,B) MJS-A2-pp65 cells were lentivirally transduced to express gp150 or control GFP (vector). Ag-presenting cells expressing low or high levels of gp150 protein were obtained by harvesting the floating cells and by FACS-sorting the adherent GFP^+^ fraction into GFP^int^ and GFP^high^ cells. Following overnight co-culture of these cells with either A2/pp65-specific CD8^+^ (A) or DR3/MiHa-specific CD4^+^ T cells (B), IFN-γ secretion into the culture supernatants was determined by ELISA. Relative T cell recognition is depicted with IFN-γ levels produced in response to stimulation with vector control cells set at 100%. C) 293T-K^b^ cells were transfected with an empty vector control or gp150. Cells were pulsed with SIINFEKL or a control peptide (SSIEFARL) and stained with Ab 25D1.16, which is specific for K^b^/ SIINFEKL complexes. D) Statistical analysis of three independent experiments (performed as in C)) using the Student’s t-test (two-tailed).

To assess if EBV gp150 expression in Ag-presenting cells affects T cells activation, the capacity of HLA-A2^+^DR3^+^ MJS-A2 cells to induce interferon (IFN)-γ production was monitored with well-defined T cell clones with different specificities. Activation of CD8^+^ T cells specific for a human cytomegalovirus (HCMV) pp65 epitope presented in the context of HLA-A2 was analysed after stimulation with pp65-expressing MJS-A2 cells that did or did not co-express EBV gp150 (Fig 3A). Whereas GFP^+^ control cells efficiently activated the T cells, IFN-γ production was markedly reduced in T cells cultured with gp150-expressing Ag-presenting cells (Fig 3A). The use of another T cell clone, a CD4^+^ T cell directed against a minor histocompatibility Ag (MiHa) endogenously expressed by MJS cells and presented in the context of HLA-DR3, allowed us to study inhibition of T cell responses restricted by HLA II. Diminished secretion of IFN-γ by the T-helper cells was observed in response to cells expressing gp150 compared to control cells (Fig 3B). These results show that not only detection of Ag-presenting molecules by Ab was blocked by gp150, but also T cell activation was hampered.

T cell activation upon target cell engagement involves formation of multiple receptor-ligand pairs, including TCR–HLA/Ag as well as costimulatory and adhesion molecule interactions. In view of the broader target range of EBV gp150, it could diminish multiple of these interactions. Here, we zoomed in on the effect of gp150 on MHC/peptide–TCR interactions. To this end, human 293T cells were used that express the mouse MHC molecule H-2K^b^ to present the ovalbumin-derived SIINFEKL epitope. Display of SIINFEKL-loaded K^b^ molecules at the cell surface was detected with the 25D1.16 Ab, thereby recapitulating TCR binding (Fig 3C). Upon introduction of EBV gp150 into these cells, a significant reduction in surface levels of epitope-loaded K^b^ molecules was observed (Fig 3C and 3D). This further supports the conclusion that T cell activation is diminished upon gp150-induced downregulation of surface Ag-presenting complexes.

Our combined results indicate that EBV gp150 impairs Ag presentation to both HLA I- and II-restricted T cells.

### EBV gp150 does not induce the degradation of Ag-presenting molecules

**Fig 4:**
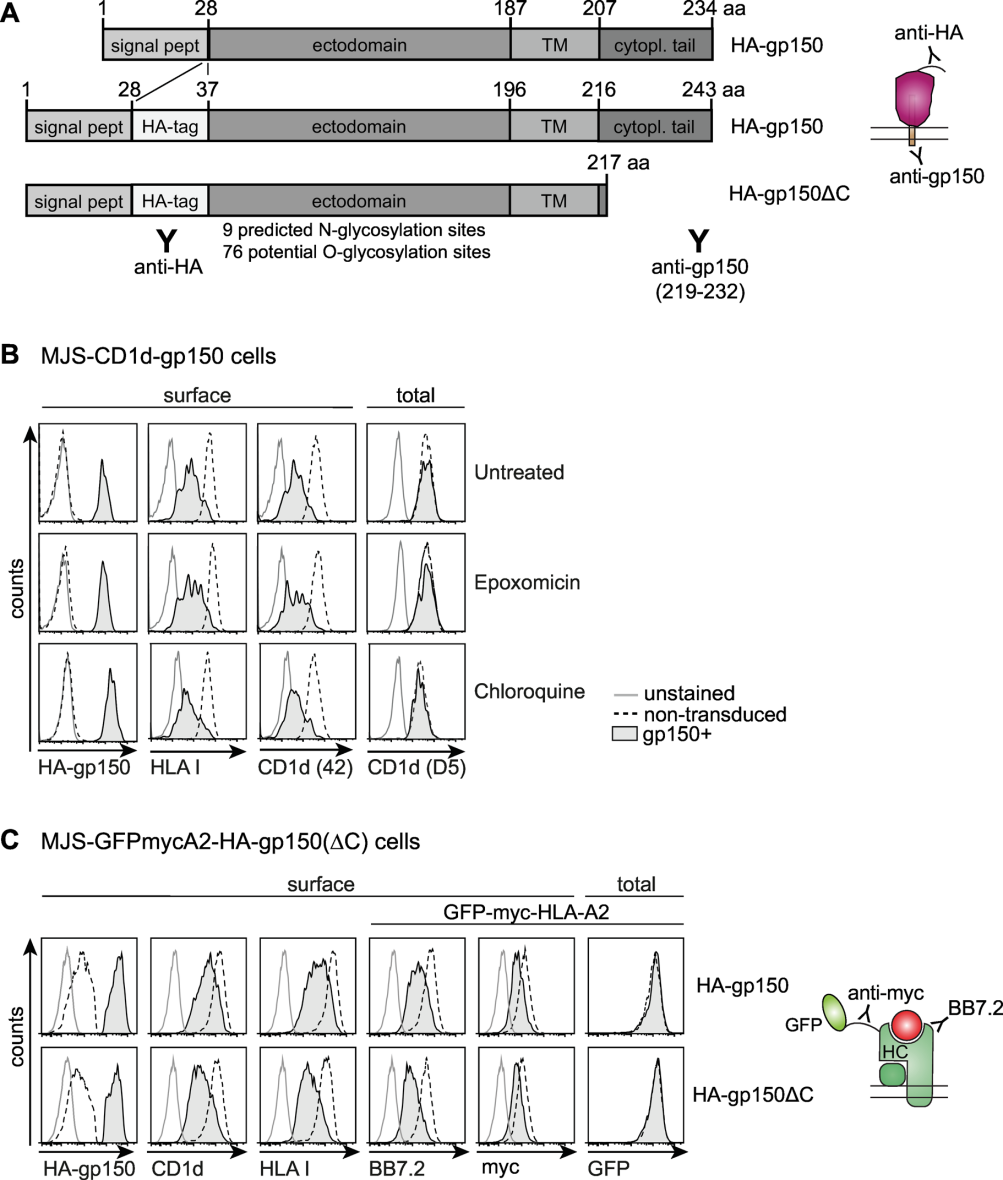
Immune evasion by EBV gp150 does not depend on degradation of Ag-presenting molecules nor on gp150’s cytoplasmic tail. A) Schematic overview of the gp150 constructs and Abs used. Signal peptide, transmembrane domain, and cytoplasmic tail are abbreviated as signal pept, TM, and cytopl tail, respectively. B) Three days after lentiviral transduction, MJS-CD1d-gp150 cells were cultured overnight in the presence of an inhibitor of proteasomes (15nM epoxomicin) or of endolysosomal proteolysis (25μM chloroquine). Subsequently, flow cytometry was used to determine surface levels of HA-tagged gp150 (HA-gp150; detected with anti-HA Ab), HLA I, and mature CD1d (Ab #42) complexes on non-permeabilized cells and intracellular staining was performed to detect immature CD1d (Ab D5) molecules in permeabilized cells. C) MJS-GFPmycA2 cells were transduced with a lentivirus encoding HA-gp150 (upper) or HA-gp150ΔC (lower). Cell surface levels of CD1d, HLA I, and HLA-A2 (BB7.2 and anti-myc tag) and total HLA-A2 (GFP) levels were compared for gp150^-^ and gp150^+^ cells.

Having demonstrated the functional impact of gp150-induced T cell evasion, we wanted to unravel its mechanism of action. In the remainder of these studies, we focused on the adherent fraction of gp150-expressing cells, although the effects might even be stronger for floating cells. In addition, we made use of constructs that encode gp150 with an N-terminal HA-tag (Fig 4A) to allow visualisation of the proportion of this EBV molecule expressed at the cell surface.

First, we investigated if reduced surface display of Ag-presenting molecules was due to gp150-induced protein degradation. Degradation of HLA molecules either by proteasomes residing in the cytoplasm or by endolysosomal proteases is an evasion strategy exploited by a number of herpesviruses, for instance by HCMV and by Kaposi’s sarcoma herpesvirus [4]. For EBV gp150-induced effects, however, no difference in surface levels was observed for HLA I or CD1d in the presence either of the proteasome inhibitor epoxomicin or of chloroquine, an inhibitor of the endolysosomal degradation pathway (Fig 4B). Of note, HA-gp150 molecules arrived at the cell surface, irrespective of treatment with these inhibitors. Additionally, intracellular staining with the D5 Ab, which detects free CD1d heavy chains (not associated with β_2_m) [20], revealed no reduction in this intracellular population of CD1d molecules in the presence of gp150 (Fig 4B, right panels), suggesting that gp150 does not induce degradation of newly synthesized CD1d molecules.

In an alternative approach, we compared gp150’s effect on surface display directly to that on total cellular levels of HLA I in the form of an N-terminally eGFP-myc-tagged HLA-A2 molecule [21], which was introduced into the HLA-A2^-^ cell line MJS (MJS-GFPmycA2 cells). In the non-permeabilized cells, GFP fluorescence reflected total levels of this fusion protein and, at the same time, surface levels were visualized with Abs directed to the extracellular myc-tag or HLA-A2 epitope (BB7.2). High levels of BB7.2 and anti-myc Ab staining as well as GFP fluorescence were detected in MJS-GFPmycA2 cells by flow cytometry (Fig 4C, non-transduced cells), indicating that the HLA-A2 fusion protein was properly expressed and reached the cell surface. Upon subsequent introduction of HA-gp150, we observed a strong reduction in surface detection of the chimeric HLA-A2 molecule, whereas total GFP levels remained unaffected (Fig 4C, gp150^+^ cells). These data implicate that in gp150-expressing cells total levels of HLA molecules remain unaltered, yet their surface detection is reduced.

In the same experimental set-up, we studied which part(s) of gp150 are essential for immune evasion. EBV gp150 is a type I membrane protein with a short cytoplasmic tail (Fig 4A). Some herpesvirus proteins, such as EBV BILF1, depend on their cytoplasmic tail for interference with Ag presentation [22]. As deletion of 26 amino acids from the C-terminus of gp150 (HA-gp150ΔC) did not impair the apparent downregulation of HLA I (endogenous and A2-GFP; Fig 4C, lower panels) or of CD1d from the cell surface, we conclude that the immune evasion function of gp150 is independent of its cytoplasmic tail.

The combined data obtained upon protease inhibitor treatment and with the GFPmycA2 fusion protein demonstrate that gp150 does not promote degradation of Ag-presenting molecules as an immune evasive strategy.

### EBV gp150 prevents detection of Ag-presenting molecules at the cell surface

Although the Ag-presenting molecules were not degraded in gp150^+^ cells, they were not detectable at the cell surface either. Therefore, we probed the intracellular location of these molecules both in gp150-expressing cells and controls.

**Fig 5:**
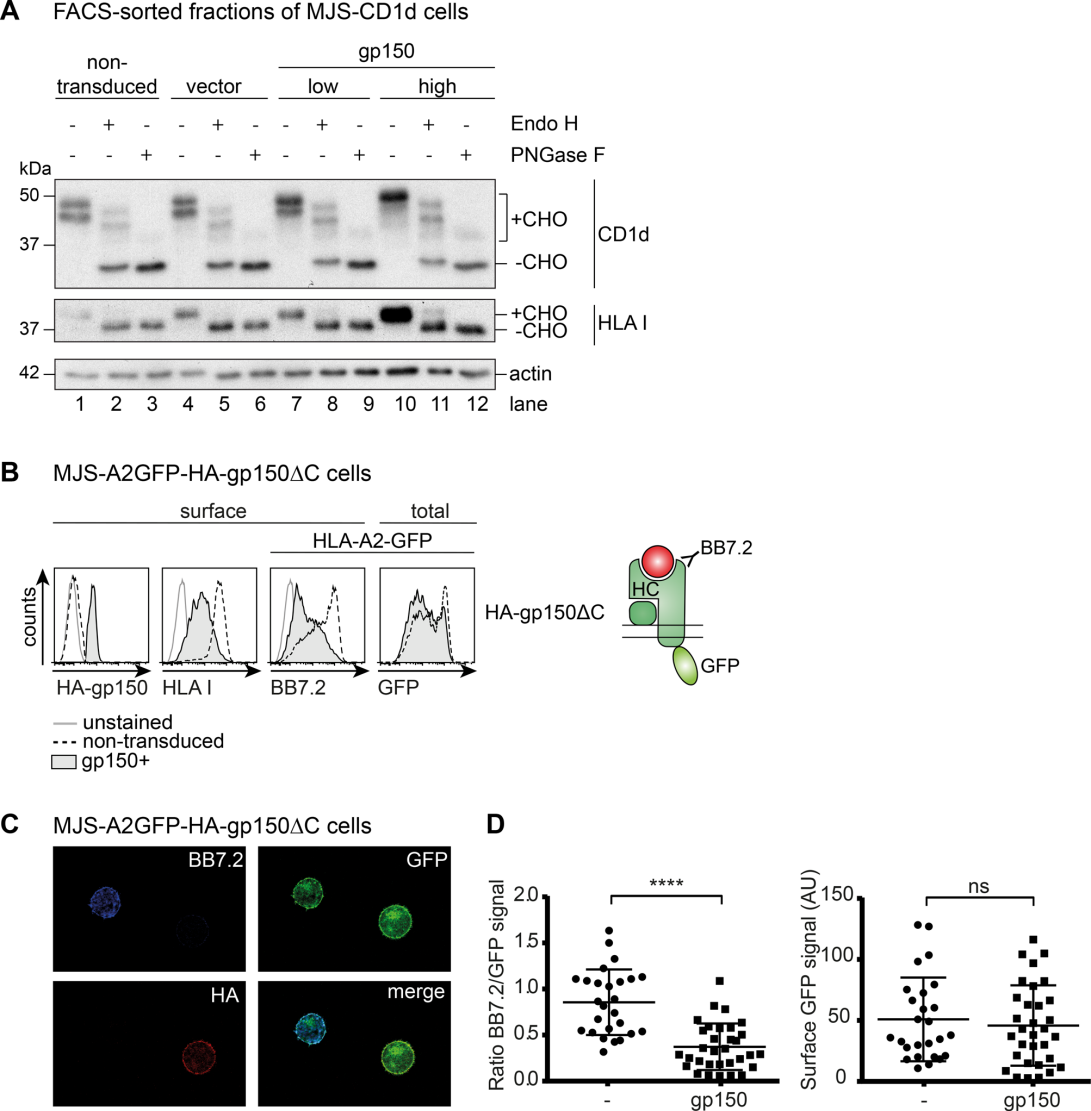
Immune evasion by EBV gp150 occurs at the cell surface. A) The glycosylation status of CD1d and HLA I molecules in lysates of MJS-CD1d-gp150 and control cells was analysed by Western blot. To this end, denatured post-nuclear lysates of different FACS sorted cell populations (see S4 Fig) were treated with Endo H or PNGase F to remove N-linked glycans. Endo H digestions served to examine protein transport beyond the cis-Golgi compartment; PNGase F digestions revealed the deglycosylated protein backbone (minus carbohydrates, -CHO). Actin was a loading control. B) Using the same experimental setup as in Fig 4C, MJS-A2-GFP cells were transduced with a lentivirus encoding HA-gp150ΔC. Cell surface levels of CD1d, HLA I, and HLA-A2 (BB7.2) and total levels of HLA-A2 (GFP) were compared for (non-permeabilized) gp150^-^ and gp150^+^ cells. C) Confocal microscopy of non-permeabilized MJS-A2GFP-HA-gp150ΔC cells. Cell surface stains were performed for HLA-A2 (BB7.2) and gp150ΔC (HA). D) Quantification of surface expression of the indicated molecules based on multiple microscopy pictures.

As trafficking of newly synthesized glycoproteins from the ER onwards is reflected by the progressive maturation of their glycans, we compared the migration of the CD1d and HLA I heavy chains in Western blots using lysates of sorted populations of control and gp150-transduced cells (non-transduced, vector, and gp150^low/high^, respectively; see S4 Fig). Differentially glycosylated maturation forms of these Ag-presenting molecules were identified upon glycosidase treatment. Endoglycosidase H (Endo H) removes high-mannose N-linked glycans, but not complex glycans that arise after the transport of glycoproteins beyond the cis-Golgi compartment. Peptide N-glycosidase F (PNGase F) treatment reveals the deglycosylated protein backbone, as it removes all N-linked glycans regardless of their maturation. Mature CD1d heavy chains carry 4 N-linked glycans, three of which become Endo H-resistant and one of which remains Endo H-sensitive [23]. In untreated control cells, two CD1d bands were detected that probably reflect different glycosylation stages of CD1d (Fig 5A,+CHO, lanes 1 and 4). PNGase F treatment resulted in one dominant band and a very faint band, reflecting virtually complete deglycosylation of CD1d heavy chains (-CHO, lanes 3 and 6). Endo H treatment revealed the deglycosylated CD1d heavy chain (-CHO, lanes 2 and 5); the two additional bands represent Endo H-resistant heavy chains, showing that these CD1d molecules have travelled beyond the cis-Golgi.

The HLA I heavy chain carries a single N-linked glycan and only one band is observed in untreated control cell lysates (lanes 1 and 3), which is removed upon digestion with both PNGase F (lanes 3 and 6) and Endo H (lanes 2 and 5). Only a minor fraction of the HLA I molecules apparently becomes Endo H-resistant in these MJS cells.

The pattern of maturation forms of CD1d and HLA I in gp150^+^ cells was virtually identical to that observed in control cells (compare lanes 1–6 to 7–12). If anything, the mature, Endo H-resistant heavy chains were more prominent in gp150^+^ cells. From these data, we conclude that the ER-to-Golgi trafficking of new HLA I and CD1d molecules is not inhibited in the presence of EBV gp150. Furthermore, the presence of the heavily glycosylated viral protein in the ER does not appear to act as a “glycosylation sink” preventing the exit of newly synthesized Ag-presenting molecules.

Confocal microscopy allows the visualisation of Ag-presenting molecules within the cell as well as their potential colocalization with EBV gp150 [22]. For these experiments, we employed non-permeabilized, non-fixed MJS cells expressing a C-terminal GFP fusion molecule of HLA-A2 (A2GFP), which had been used before in microscopy studies on HLA I maturation and trafficking [24]. Flow cytometry analysis confirmed that this HLA-A2 fusion protein behaved similarly to the other Ag-presenting molecules tested. Introduction of gp150 in MJS-A2GFP cells decreased surface detection by HLA I-specific Abs, while GFP fluorescence indicated that the total amount of cellular A2GFP molecules was not altered by gp150 (Fig 5B). In confocal microscopy, gp150^+^ MJS-A2-GFP cells were identified based on positive staining for the HA-tag attached to gp150 (in red; Fig 5C, lower left panel), which confirmed that HA-gp150 molecules arrived at the cell surface. Focusing on the non-transduced (HA^-^) control cells, the GFP signal shows localization of A2GFP fusion proteins at the plasma membrane (Fig 5C, upper right panel). Accordingly, the surface-exposed A2GFP molecules were detectable with Ab BB7.2 (Fig 5C, upper left panel), yet only in the non-transduced fraction of cells. This costaining demonstrated the integrity of the chimeric HLA-A2 molecule. Using Ab-staining (BB7.2), surface A2GFP molecules were not detected on the gp150^+^ fraction of cells by confocal microscopy (Fig 5C, upper left panel), as may have been anticipated from the flow cytometry data described above (Figs 1, 2 and 4). However, GFP fluorescence revealed the presence of the A2GFP molecules also at the surface of gp150^+^ cells, where they colocalized with gp150 (Fig 5C, right panels). The arrival of HLA I molecules at the surface of both gp150^+^ and control cells, indicates that gp150 does not alter the localization of these Ag-presenting molecules. Quantification of multiple microscopy pictures supports that the Ab-based detection of HLA-A2-GFP is significantly reduced for gp150-expressing cells compared to control cells and this was not due to reduced GFP fluorescence intensity at the cell surface (Fig 5D).

Together, these data indicate that cellular expression of EBV gp150 does not alter the total protein levels or localization of HLA molecules, but prevents their Ab-based detection on the cell surface.

### EBV gp150 shields surface molecules via sialoglycans on its ectodomain

Since the cytoplasmic tail of gp150 is not essential for immune modulation, it is likely that the extracellular domain of gp150 is mechanistically involved. The most peculiar characteristic of EBV gp150 is its highly abundant glycosylation, with about half of the amino acid residues in the N-terminal domain being potential substrates for N- or O-linked glycosylation (Fig 4A). Its extensive glycosylation is reflected by the observation that in SDS-PAGE gels, gp150 migrates as a diffuse band with a molecular weight of 100–150 kDa, whereas the unglycosylated protein backbone is around 30 kDa [25,26]. Although the exact glycan composition is not known, gp150 carries many negatively-charged sialic acid moieties [25,26]. To directly assess whether the glycan decoration on gp150 plays a role in the shielding of surface Ag-presenting molecules, flow cytometry analysis was performed on gp150 expressing cells from which sialic acids had been depleted using three different approaches.

**Fig 6:**
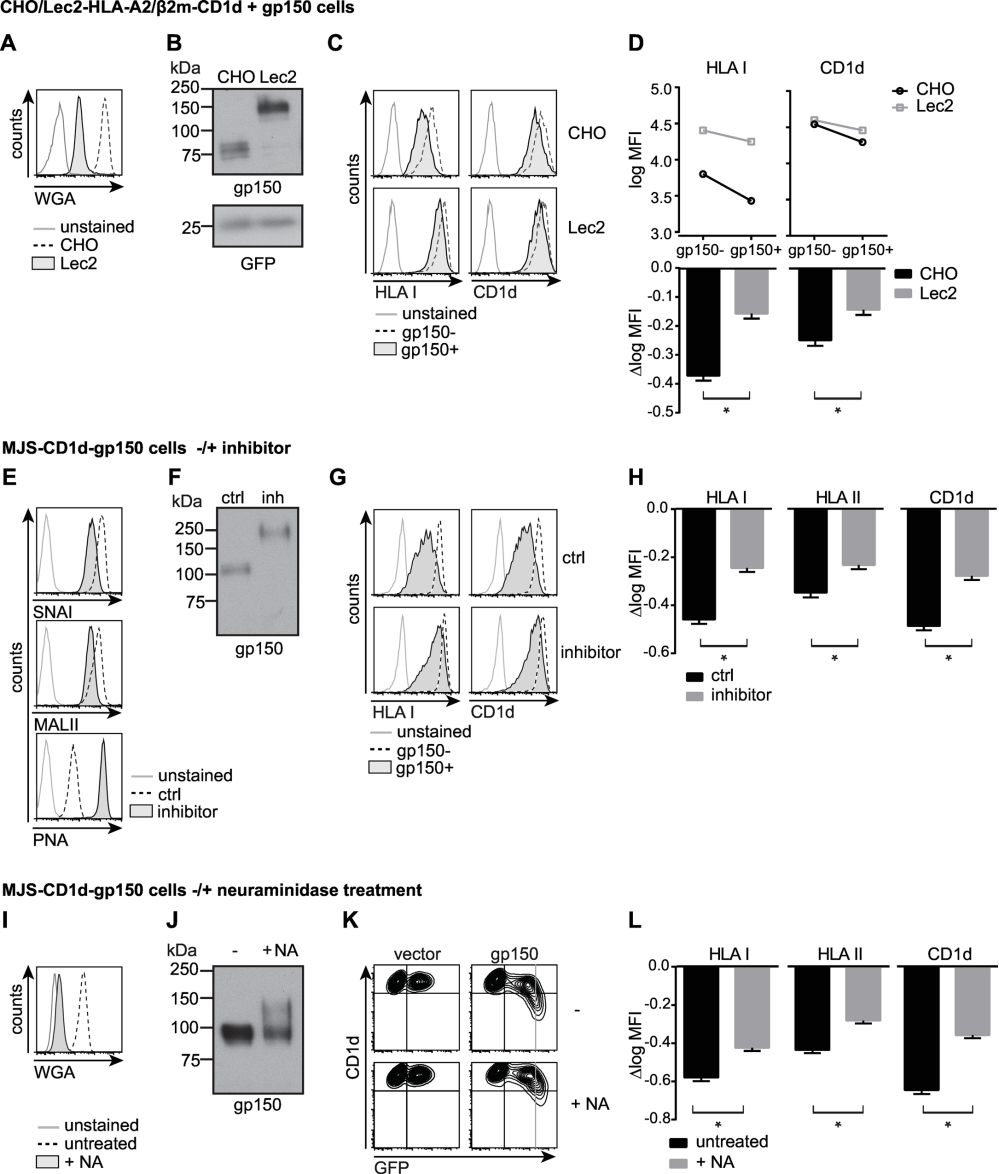
The mechanism of gp150-induced immune evasion relies on sialoglycans shielding surface-exposed Ag-presenting molecules. A-D) CHO and Lec2 cells expressing human β_2_m, HLA I, and CD1d were lentivirally transduced to co-express gp150 or HA-gp150ΔC and GFP (from EF1a and PGK promoters, respectively). A) Lectin (WGA-FITC) binding confirmed the glycosylation defect in Lec2 cells compared to parental CHO cells. B) Migration height of HA-gp150 was visualised by Western blot analysis. C-D) Levels of HLA I and CD1d at the surface of HA-gp150ΔC^+^GFP^+^ (gp150^+^) cells were compared to those on control, non-transduced GFP^-^ (gp150^-^) cells. D) Surface levels of HLA I and CD1d are depicted as log MFI values with 95% confidence intervals, for gp150^+^ versus gp150^-^ wt CHO cells or sialylation-defective Lec2 cells. The slopes of the connecting lines (Δlog MFI) reflect the downregulation induced by gp150. Statistical analysis was performed using two-way ANOVAs and significance of the interaction term was assessed, as described in the Material and Methods section. One representative experiment of at least six is depicted. * p<0.01. E-L) MJS-CD1d-gp150 cells were generated by transduction with a lentivirus encoding both gp150 and GFP (from a CMV promoter and an IRES sequence, respectively) and were analysed 3 days post-transduction by Western blot and by flow cytometry, as for A-D with the modifications indicated below. E-H) To prevent sialylation, cells were treated with the sialic acid transferase inhibitor (500 μM inhibitor) fluorinated P-3F_ax_-Neu5Ac. As a control, cells were treated with the non-fluorinated compound (500 μM, ctrl) for 4 days, starting 1 day prior to transduction. This control treatment was comparable to when cells were left untreated. E) Lectins SNAI, MALII, or PNA were used to detect sialoglycans or desialylated glycans, respectively. G) gp150^+^GFP^high^ cells were compared to non-transduced cells. H) One representative experiment of two is depicted. * p<0.01. I-L) To remove surface sialylation, intact cells were treated with neuraminidase (1U/μl, 60 min, 37°C) prior to cell lysis or lectin/Ab staining. L) One representative experiment of three is depicted. * p<0.01.

First, we introduced human CD1d, HLA-A2, and β_2_m both into Lec2 cells, which are Chinese hamster ovary (CHO) cells with a mutated CMP-sialic acid Golgi transporter resulting in decreased sialylation of glycans [27], and into control CHO cells. Defective sialylation in the Lec2 cells was confirmed by decreased binding of wheat germ agglutinin (WGA), a lectin that binds sialic acid and *N*-acetylglucosamine structures (chitobiose) (Fig 6A). The effect of defective glycosylation on EBV gp150 expressed in Lec2 cells was visible by Western blot: compared to lysates from control CHO cells, gp150 from Lec2 cell lysates migrated at a higher apparent molecular weight, likely due to the absence of negatively charged sialic acids (Fig 6B). Staining for surface HLA I and CD1d on Lec2 cells was somewhat increased compared to parental CHO cells, probably reflecting a better accessibility of the Ab binding sites when the Ag-presenting molecules are less sialylated (Fig 6C and 6D). In the CHO cells, expression of EBV gp150 decreased HLA I and CD1d staining, which was partly reversed in Lec2 cells. This indicates that sialic acids contribute to the phenotype caused by gp150 (Fig 6C and 6D).

Second, to address the role of sialylation in protein downmodulation in human cells and, thereby, also to be able to test a broader panel of surface markers, sialylation was inhibited by pretreating MJS-CD1d cells with the fluorinated sialic acid analogue P-3F_ax_-Neu5Ac [28]. This analogue blocks sialyltransferases, enzymes that transfer sialic acids onto glycans. Inhibition of sialylation was detected by the reduced binding of the sialic acid-binding lectins SNAI and MALII to treated cells (Fig 6E). Strongly enhanced binding of PNA, a lectin that binds to glycan structures that become accessible upon desialylation, further confirmed successful inhibition of sialyltransferases following cell culture in the presence of this competitive inhibitor (Fig 6E). Similar to the sialylation-defective hamster cells, sialyltransferase inhibitor treatment of MJS-CD1d cells expressing gp150 was sufficient to cause the viral protein to migrate differently in SDS-PAGE (Fig 6F). Cells transduced to express gp150 that were pre-incubated with P-3F_ax_-Neu5Ac showed increased surface staining for HLA I, II, and CD1d compared to control cells (Fig 6G and 6H), consistent with a crucial role for sialylation in the shielding of Ab binding sites by gp150.

Third, to selectively cleave off cell surface sialic acids, we treated cells with neuraminidase. Enzymatic desialylation occurred in a dose-dependent fashion, as witnessed by progressive loss of WGA lectin binding at the cell surface (Figs 6I and S5). Selectivity of this neuraminidase treatment for surface-exposed glycans was supported by Western blot analysis, in which a proportion of gp150 molecules from treated cells displayed reduced mobility, whereas the remaining, likely intracellular, EBV proteins had an apparent molecular weight comparable to that in untreated cells (Fig 6J). At the surface of gp150-transduced MJS-CD1d cells, CD1d detection improved with increasing doses of neuraminidase (Figs 6K and S5), pointing to a direct involvement of sialic acid-modified glycans in interference with CD1d detection. Likewise, surface display of the host cell molecules HLA I and HLA II was improved by neuraminidase treatment (Fig 6K and 6L) to the same range as observed for the inhibitor treatment (Fig 6G and 6H), suggesting that primarily sialic acids at the cell surface played a role.

In the three settings above (Fig 6), a rescue of 15–25% in the surface detection of Ag-presenting molecules was observed in the presence of desialylated versus fully sialylated gp150. This rescue being modest is perhaps due to the remaining glycan structures on gp150. Still, a highly significant increase in display was detected in all abovementioned experimental approaches. Therefore, these combined results provide evidence that surface sialic acid-carrying N- and O-linked glycans contribute to EBV gp150’s ability to hide certain host cell surface molecules, including those involved in Ag presentation, thereby providing a mechanistic basis for T and iNKT cell evasion.

### EBV gp150 mediates glycan shielding of Ag-presenting molecules on human B cells

Natural EBV infection *in vivo* targets human B cells and epithelial cells and the main reservoir of persistent infection is the memory B cell. To produce new offspring, EBV reactivates from latently infected B cells carrying the viral genome. Whereas the 293T and MJS cells could reflect epithelial infection, we specifically addressed the question if gp150-mediated immune evasion was also operative in human B cells.

As relevant human B cells for this study, we chose Akata BL cells in latency, which upon induction are capable of supporting productive EBV infection, implying that all host cell factors essential for EBV replication are present. These Akata BL cells are the parental equivalent of the AKBM cells described above (without the rat CD2GFP lytic cycle reporter). In the Akata BL background, Borza and Hutt-Fletcher [19] had previously generated a knockout mutant EBV that lacks the *BDLF3* open-reading frame coding for gp150 (AkataΔgp150 cells, see below). For immune evasion studies, we introduced a constitutively expressed EBV gp150 gene into these latent AkataΔgp150 B cells, thereby avoiding potential interference by any endogenous gp150 expression (referred to as Akata+gp150 cells).

**Fig 7:**
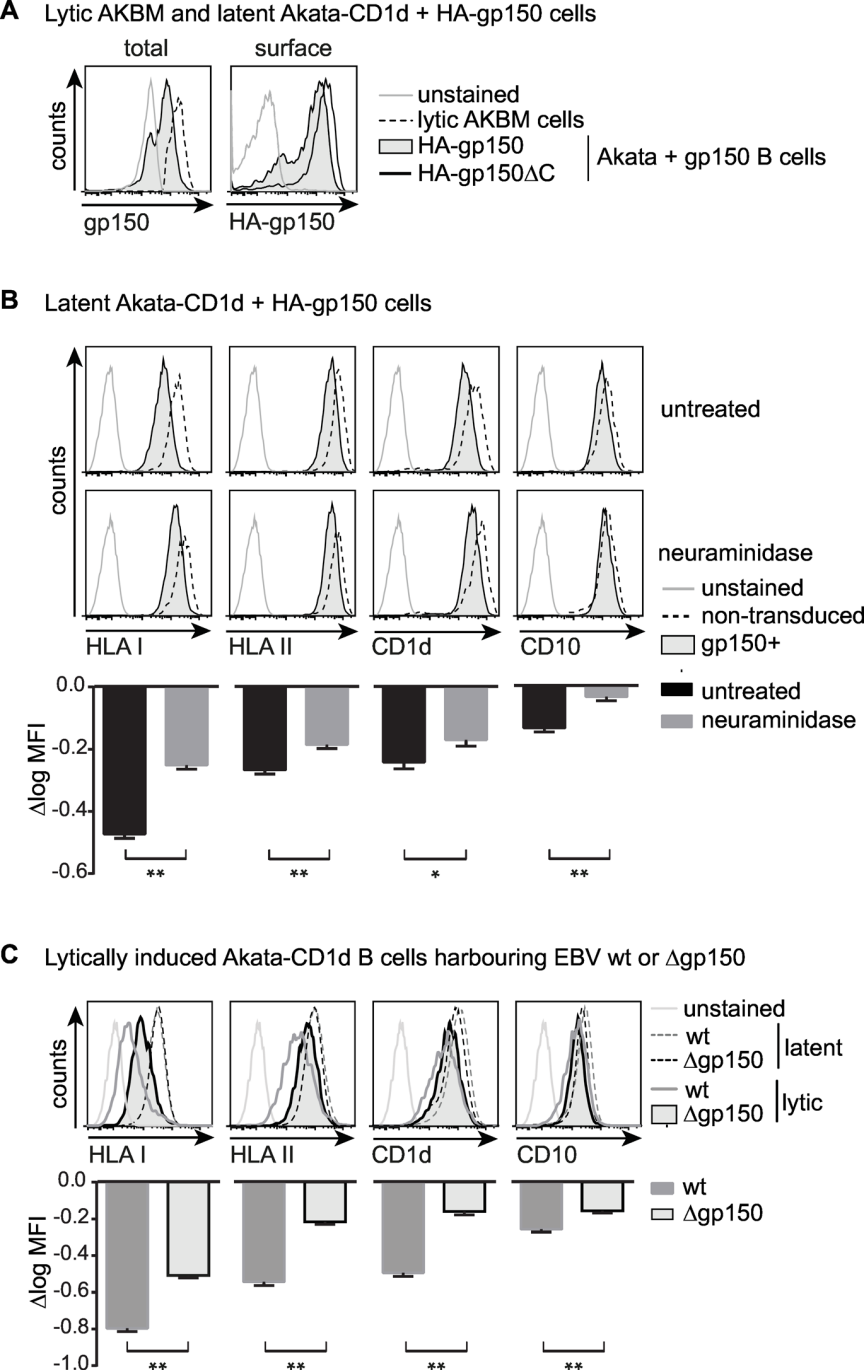
Glycan shielding of surface Ag-presenting molecules by gp150 occurs in human B cells and is reversed during productive EBV infection when gp150 is deleted. A-B) Latent AkataΔgp150 cells were lentivirally transduced to co-express either HA-gp150 or HA-gp150ΔC and GFP (from EF1a and PGK promoters, respectively) and were puromycin-selected to obtain pure populations of gp150^+^ cells. B cells already grow in suspension and gp150-positive B cells were maintained in culture for several weeks, indicating that gp150 expression was not toxic to the cells. A) Flow cytometry was used to assess total (intracellular staining with anti-gp150 Ab on permeabilized cells) and surface (anti-HA Ab on non-permeabilized cells) levels of EBVgp150. Expression levels of gp150 were compared to lytically induced AKBM cells (20 hours anti-human IgG treatment, rat CD2GFP^+^ cells). B) Akata+gp150 and non-transduced control cells were left untreated or were treated with neuraminidase (1U/μl, 60 min, 37°C). Surface levels of Ag-presenting molecules and cellular CD10 as a control were compared to gp150^-^ non-transduced cells. One representative experiment of three is depicted. C) Viral reactivation was induced in Akata wt and Δgp150 B cells by overnight culture with anti-human IgG and EBV-producing cells were identified by staining for the late viral protein gp350. Surface levels of Ag-presenting molecules and CD10 on lytic (gp350^+^) and latent (gp350^-^) cells are depicted in overlay histograms. One representative experiment of at least four is depicted. Statistical analysis was performed for B and C) as described for Fig 6. * p<0.05, ** p<0.01.

To achieve gp150 expression at levels similar to those occurring in EBV-producing B cells, we compared gp150 protein levels in Akata cells transduced with HA-gp150 or HA-gp150ΔC lentiviruses to those in lytically induced AKBM cells. In flow cytometry using an Ab against the gp150 C-terminus, levels of gp150 in productively infected B cells appeared to exceed those in cells transduced to express full-length gp150 in isolation (Fig 7A, left panel). The C-terminally truncated gp150 lacks the epitope recognized by the anti-gp150 Ab. Therefore, we next compared full-length and truncated gp150 expression levels in transduced B cells by means of their HA-tag and found that levels of the cytoplasmic tailless gp150 were slightly higher than those of the full-length protein (Fig 7A, right panel). Based on this, we reasoned that B cells expressing gp150ΔC in isolation likely reflect the levels occurring in the context of natural EBV-infection of B cells and we have, therefore, studied these cells in more detail.

Detection of HA-tag on non-permeabilized HA-gp150 Akata cells (Fig 7B) also shows that the gp150 protein arrives at the surface of B cells as it did on 293T (Fig 1C) and MJS (Figs 2A, 4B and 4C, 5B and 5C) cells. At the surface of HA-gp150ΔC-transduced Akata B cells, detection of HLA I, II, CD1d, and to a lesser extent CD10 was reduced in comparison to untransduced control cells (Fig 7B, upper panels). This shows that gp150 induces an immune evasive phenotype in human B cells as it did in the other cell types studied. Neuraminidase treatment of Akata+gp150 cells increased surface binding of Abs specific for the three Ag-presenting molecules and CD10 (Fig 7B, lower panels and bar graphs), pointing towards direct involvement of sialic acids in gp150-induced shielding of B cell surface markers.

These data combined with those in Fig 6 of gp150 masking surface HLA I, II, and CD1d via a glycan shield indicate that the same mechanism of action can operate both in epithelium-like cells and in B cells, the natural targets for EBV infection *in vivo*.

### EBV gp150 contributes to evasion of Ag presentation during productive infection

To assess the contribution of gp150-mediated interference with Ag presentation in the full viral context, we studied the productive phase of infection in B cells that harbour either wild-type EBV (Akata wt) or the *BDLF3* deletion mutant AkataΔgp150. Based on the use of these cells, gp150 was earlier reported not to be essential for EBV replication *in vitro*, as the recombinant virus had no defects in binding, infectivity, assembly, or egress [19]. In line with this, we found no apparent differences in progression through the replicative cycle between lytically induced Akata wt and Δgp150 cells: both expressed the EBV immediate-early BZLF1, early BGLF5, and late gp350 proteins, but only Akata wt cells produced gp150, as did control induced AKBM cells (S6A Fig).

To determine surface levels of the host cell molecules HLA I, II, CD1d, and CD10 by flow cytometry, both types of induced Akata cells were counter-stained for gp350, to identify the EBV-producing B cells. As observed for AKBM cells (Fig 1A), induction of the lytic cycle caused a downregulation of surface display of the Ag-presenting molecules HLA I, II, and CD1d in (gp350^+^) Akata wt cells, when compared to the latently infected cells (Fig 7C). In the absence of gp150, however, HLA I, II, and CD1d surface levels were significantly higher during productive infection, as visible when comparing gp350^+^ AkataΔgp150 to Akata wt cells (Fig 7C, compare tinted with non-filled grey histograms). Likewise, downregulation of the B cell surface markers CD10 and CD86 was less pronounced in lytic cells devoid of gp150 (Fig 7C and S6B Fig). These results indicate that gp150 contributes to the apparent downregulation of Ag-presenting molecules and–to a lesser extent- other surface markers in a model for natural EBV infection of human B cells.

**Fig 8:**
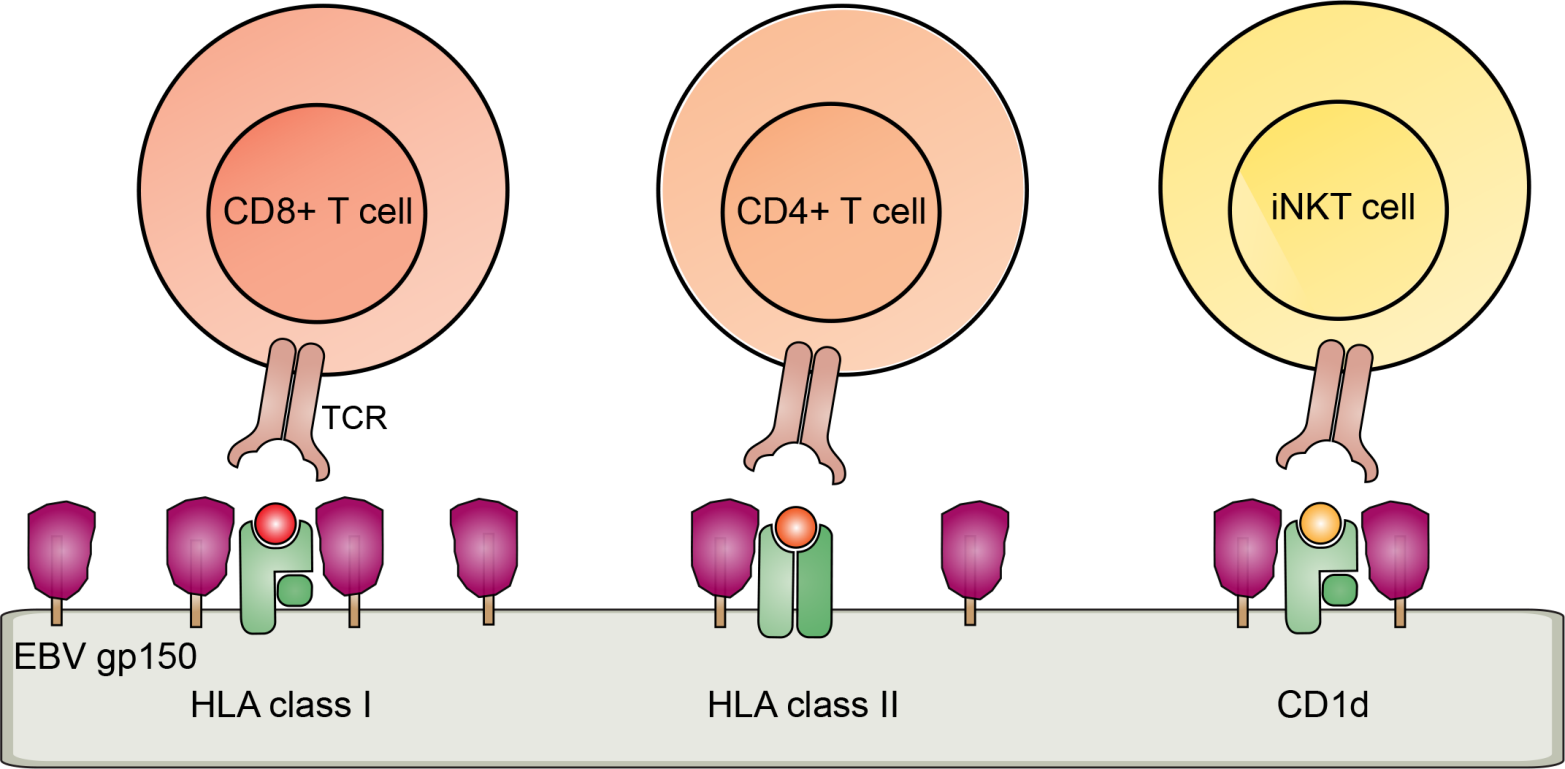
Schematic model of a glycan shielding mechanism for T and iNKT cell immune evasion mediated by EBV gp150.

Our combined data support a model in which surface molecules, including Ag-presenting molecules, are shielded from recognition by the extensive glycosylation of gp150, pointing towards a contribution of this novel EBV evasion molecule to interference with cell-mediated immunity against EBV (Fig 8).

## Discussion

Cell-mediated immunity by HLA I and II-restricted cytotoxic and helper T cells as well as by CD1d-restricted iNKT cells is critical in the defence against many viruses [1]. Especially persistent viruses, such as herpesviruses, have acquired strategies to permit their escape from elimination by these immune cells. In this study, we report that EBV infection, even in its late productive phase, substantially reduced display of CD1d, HLA I, and HLA II complexes at the surface of human B cells. Reduced detection of these antigen-presenting molecules was observed with Abs that can block TCR binding. This effect is functionally important, because HLA downregulation results in reduced T cell activation. For transmission to other target cells or hosts, new viral particles need to be produced through EBV reactivation from latently infected B cells, the primary natural targets for EBV infection. At the late stage of viral replication, up to 100 EBV genes are expressed, thus resulting in a wide array of Ags presented at the surface of infected cells to T cells. EBV-specific T cells are generated upon primary infection [6]. Therefore, immune escape strategies targeting Ag-presenting molecules including HLA I, II, and CD1d might allow EBV-producing B cells to effectively evade T and iNKT cell surveillance *in vivo*, prolonging the timespan for the production of viral offspring. Indeed, patients with reduced functional T and iNKT cell numbers are known to have life-threatening complications upon encountering EBV [6].

Our finding that gp150 acts as a novel EBV-encoded immune evasion molecule causing reduced detection of surface CD1d, HLA I, and HLA II complexes is intriguing as it is not only the first function ascribed to this viral glycoprotein, it also reveals a novel mechanism employed by herpesviruses to dodge immune recognition. In contrast to most herpesvirus structural glycoproteins, EBV gp150 was found to be non-essential for viral entry, assembly, or egress [19] and the function of this late viral protein has remained enigmatic until now. Here, we provide evidence in support of a model in which gp150 is able to shield surface HLA molecules, through its abundantly sialylated glycans, in order to escape T cell activation (Fig 8). Cellular expression of gp150 in isolation was sufficient to reduce detection of multiple cellular surface molecules, for example interfering with binding of an Ab detecting the H-2 K^b^/SIINFEKL antigenic complex. Moreover, we show that B cells producing a gp150-deleted mutant virus display more Ag-presenting molecules as compared to wild-type EBV, indicating that gp150 contributes to immune evasion in the full viral context. EBV gp150 did not appear to promote degradation, retention, internalization, or altered glycosylation of HLA molecules. Rather, HLA molecules arrived at the cell surface, yet failed to be recognized, a defect which was restored in part by preventing sialylation.

To date, only the Ebola virus glycoprotein (EBOV GP) has been described to shield host cell surface molecules, namely HLA I and β1 integrin molecules, via its extensively glycosylated mucin-like domain [29]. This suggests that similar mechanisms have evolved independently in different viruses belonging to different families. Both EBOV GP’s mucin-like domain and the ectodomain of EBV gp150 contain around 10 potential N-glycosylation and more than 70 potential O-glycosylation sites. The mucin-like domain of EBOV GP consists of mainly core 2 O-linked glycans [30]. The ‘core’ type of O-linked glycans on gp150 is, at present, unknown, but could also consist of core 2 glycans in view of its resistance to O-glycanase that removes core 1 and core 3 O-glycans [25]. Thus, it remains to be investigated whether the glycans of EBV gp150 and EBOV GP share key features or whether they evolved similarly to shield host surface molecules. Detailed characterization of the glycans of EBOV GP and EBV gp150 is likely required to fully comprehend the molecular necessities of viral glycoproteins to shield cell surface molecules and thereby facilitate further identification of such proteins.

It would be interesting to know whether other (herpes)viruses have incorporated similar strategies to escape from immune recognition, as this would provide further insight into the significance and requirements of this mechanism. The *BDLF3* ORF is also present in Rhesus LCV. Rhesus LCV BDLF3 protein displays only 46% amino acid homology to EBV gp150, but shares the feature of the serine/threonine-rich ectodomain indicating that it too might be heavily glycosylated. In view of the low sequence homology between the BDLF3 proteins of these closely related γ-herpesviruses, glycosylation potential rather than amino acid sequence might provide immune evasive properties. It will be interesting to analyse the immune evasive properties of MHV-68 ORF28, the positional homologue of EBV *BDLF3*. MHV-68 ORF28 also appears to be non-essential for viral replication *in vitro* and *in vivo* [31]. Compared to gp150, the ectodomain of ORF28 is reduced in size, yet highly glycosylated and could therefore provide insights into the structural requirements for glycan shielding. It is tempting to speculate that other proteins or different (herpes)viruses present functional homologues of EBV gp150 and EBOV GP that do not share amino acid similarity, yet retain the feature of a heavily glycosylated extracellular domain. Intriguingly, we observed that the major envelope glycoprotein of EBV, gp350, also interfered with surface detection of CD1d (Fig 1B) and TfR (S2B Fig).

Interestingly, not only do viruses appear to exploit a glycan shielding strategy, but also cancer cells show aberrant expression of sialic acid-modified glycans that are thought to play a role in tumor immune evasion [32], which may in part be due to the reported sialylation-dependent inhibition of NK cells by masking ligands of activating NK cell receptors [33]. Relevance of sialylation-dependent immune interference by tumors is demonstrated by the fact that hypersialylation of tumor cells is associated with poor prognosis for patients [34]. Interestingly, when sialic acid decoration on tumor cells was inhibited with the same compound as we have used (Fig 6E–6H), it resulted in reduced metastases in an *in vivo* mouse model [35].

Our results show a direct effect of gp150 on the presentation of antigenic peptides to T cells. In addition to Ag-presenting molecules, EBV gp150 could also affect the function of various other cell surface molecules. Potential targets include receptor-ligand pairs involved in formation of the immunological synapse. An immunological synapse is formed at the interface of the T cell and the virus-infected cell through interactions of several receptor-ligand pairs [36]. In the centre of this synapse, HLA/Ag complexes reside, engaged by specific TCRs. Costimulatory molecules locate in proximity of the Ag-presenting molecules, and are surrounded by a ring of adhesion molecules. We have observed that cellular expression of gp150 also appears to mask the adhesion molecule CD54/ICAM1 (Fig 2A), the costimulatory molecule CD86 (S6B Fig), as well as TfR (S2B Fig), and CD10 (Fig 2A). Of note, shielding of these molecules appears to require higher levels of gp150 expression than was sufficient for shielding of these Ag-presenting molecules (S2C Fig, and Fig 2 & S3B Fig). In contrast, CD33, CD44, and CD55 were not sensitive to downregulation by gp150, even when expressed at high levels (Fig 2C), showing that gp150 does not mask all cell surface molecules. The broad target range of gp150 suggests that other receptor-ligand interactions, such as receptor-induced apoptosis by FasL-Fas interactions, or other (non)-immune cell-cell contacts could be disturbed by gp150. Intriguingly, cell adherence and cell-cell contact were lost in floating MJS cells expressing high levels of gp150 (S3A Fig), as was also observed for EBOV GP [29], suggesting that gp150 can uncouple, at least partially, actively EBV-producing B cells from environmental cues. One could speculate that the EBV-producing B cell could thus become mobile by interfering with signals that restrain B cells at a particular site in the body, allowing them to localize for instance to tonsils, facilitating viral shedding in saliva.

Besides the host cell molecules described above, expression of the heavily glycosylated gp150 protein could also shield the recognition of *viral* membrane-expressed proteins that could otherwise by recognized by EBV-specific Abs. EBV exploits the endogenous glycosylation machinery of the infected cell to form glycan structures, which are, by nature, not foreign to the immune system. When these “self-structures” are placed on a—foreign—viral protein, it could be shielded from recognition by Ab-producing B cells. In line with this, no good Abs against the extracellular part of gp150 are available. Furthermore, when the glycosylation is abundant as is the case for gp150, it could also lead to the masking of other viral proteins that are expressed at the cell/ virus surface. So far, we have not observed strongly reduced detection of gHgL, gp350, or gB on wild-type versus gp150-null EBV-producing B cells, and it would not be advantageous to viral entry if these receptors were fully shielded. Yet, both for EBOV GP [37] as well as for some herpesviruses, glycosylation-mediated evasion of Ab neutralization has been reported, for example for the proteins gN of HCMV and the bovine herpesvirus 4 (BoHV-4) gp180, which is the functional homologue of EBV gp350. Truncated HCMV gN lacking part of its glycosylated ectodomain as well as absence of BoHV-4 gp180 renders viral particles more sensitive to Ab neutralization [38,39]. In addition, cells infected with BoHV-4 lacking gp180 show increased Ab binding to viral proteins, pointing to the role of glycans in shielding viral proteins from immune recognition.

Persistent viruses dedicate a large part of their genome to modulating host antiviral activities. Among these, herpesviruses have acquired a very diverse set of strategies to downregulate Ag-presenting molecules and, thereby, T cell activation in a temporal fashion. Viral strategies include blocking protein synthesis, depletion of antigenic peptides, dislocalization to the cytoplasm followed by proteasomal degradation, re-routing and/or accelerated internalization, or steric hindrance of TCR binding [4]. In this study, we report on a novel EBV strategy: by expressing the heavily glycosylated, late EBV protein gp150, Ag-presenting molecules on productively infected cells are shielded from interaction with TCRs, thereby allowing replication and production of viral particles that would otherwise by interrupted by a cellular immune response. This mechanism is novel in herpesvirus biology, and further supports the notion that herpesviruses use very diverse ways to achieve escape not only from T cells, but also from iNKT cell immunity. Evasion from CD1d-mediated Ag presentation supports an essential role for iNKT cells in anti-EBV immunity, which was already inferred from the devastating effects of EBV infection in XLP patients. Combining multiple strategies allows them to escape various layers of immune elimination during various stages of infection. This underscores the notion that EBV, as other herpesviruses, is extremely well adapted to persist in the face of functional immunity.

## Materials and Methods

### Ethics statement

For T-helper cell assays, peripheral blood samples from patients and healthy individuals were obtained after approval by the LUMC institutional review board and written informed consent according to the Declaration of Helsinki.

### Cells

All media were supplemented with 10% FCS, 100 U/ml penicillin, and 100μg/ml streptomycin. The EBV^+^ BL cell lines AKBM [15], Akata wt, and Akata Δgp150 [19] were cultured in supplemented RPMI medium. Productive EBV infection and inhibition of late protein expression by phosphonoacetic acid were performed as previously described [22]. Human embryonic kidney (HEK) 293T, HEK 293H-2K^b^, and the human melanoma cell line MelJuSo (MJS, HLA typing A*01, B*08, DR3) and MJS-derived cell lines were cultured in supplemented DMEM medium. MJS-A2-GFP cells were obtained from Dr. E Reits [24]. Chinese hamster ovary (CHO) and Lec2 cells [40] were cultured in supplemented MEM-α medium.

### Antibodies and streptavidin conjugates

Primary antibodies used in this study were mouse α-CD1d (CD1d42, CD1d51, CD1d55, D5; a kind gift by S. Porcelli, NY, USA), mouse α-CD1d-PE (clone CD1d42, BD Pharmingen), mouse α-HLA-ABC (W6/32), mouse α-HLA-ABC-PE (W6/32, Serotec), mouse α-HLA-A2 (BB7.2), mouse α-HLA-DR (L243), mouse α-HLA-DR-PE (L243, BD Biosciences), mouse α-TfR (CD71, clone M-A721, BD Pharmingen), mouse α-CD10-PE (clone Hl10a, BD Pharmingen), mouse α-CD34-PE (clone 8G12, BD Biosciences), rat α-CD44-PE (clone IM7, BD Pharmingen), mouse α-CD54-PE (ICAM-1, clone HA58, BD Pharmingen), mouse α-CD55-PE (clone IA10, BD Pharmingen), mouse α-BZLF1 (clone BZ.1, kindly provided by Dr. M. Rowe, Birmingham, UK), mouse α-BGLF5 (clone 311H, kindly provided by Dr. J. Chen, Taipei, Taiwan), mouse α-gHgL (E1D1), rabbit α-gp150 (V8), biotinylated mouse α-gp350 (72A1), rat α-HA-tag (3F10, Roche), rat α-EBNA1-tag (1H4, kindly provided by Dr. J. Mautner), mouse α-myc-tag (9E10). Secondary antibodies used in flow cytometry were goat α-mouse-PE (eBioscience), donkey α-rat-APC or–FITC (Jackson Immunoresearch), and goat α-rabbit-PE (Jackson Immunoresearch). To detect biotinylated antibodies or lectins, streptavidin-BV421 or streptavidin-PE (both BioLegend) were used, respectively. For Western blot analysis, an HRP-conjugated goat α-rabbit (Southern Biotech) antibody was used. For confocal microscopy, goat α-mouse-Atto647 (Sigma) and goat α-rat-Alexa594 (Invitrogen) antibodies were used.

### Plasmids

Plasmids encoding His- and EBNA1 (aa435-445, recognized by 1H4 Ab)-tagged EBV glycoproteins (gM, gN, gp78, gp350, and gp150) were a generous gift from Dr J. Mautner and were described previously [41].

In this study, different lentiviral vectors were used to create third generation replication-deficient lentiviruses. Expression of the human CD1d or HLA-A2 gene fused to a zeocin resistance gene by means of the ribosomal skipping peptide P2A was driven by an EF1a promoter [22]. C-terminally Flag (DYKDDDDK)-tagged *BMRF2*, N-terminally Flag-tagged *BDLF2*, and *BDLF3* EBV genes were PCR-amplified from EBV B95.8 genome. PCR products were cloned into the lentiviral vector pLV-CMV-IRES-GFP. We generated an in-frame N-terminal HA-tagged (YPYDVPDYA) gp150 full-length variant and a mutant lacking the last 26 aa (209–234) of the cytoplasmic tail (HA-gp150ΔC). The PCR products were cloned behind an EF1a promoter into the dual promotor lentiviral vector EF1a-/pGK-GFP-T2A-puro, described elsewhere [21], and into the lentiviral vectors pLV-CMV-IRES-GFP and pLV-CMV-IRES-puro^R^. The lentiviral vector encoding eGFP-myc-HLA-A2 has been described elsewhere [21].

Codon-optimized human β_2_m was encoded in a variant of the abovementioned dual promoter lentiviral vector containing a zeocin resistance gene and mAmetrine fluorescence gene fused by means of the ribosomal skipping peptide T2A.

### Replication-deficient retroviruses and retroviral transductions

Third generation replication-deficient SIN recombinant lentiviruses were generated by PEI transfection of 293T cells with pCMV-VSVG, pMDLg-RRE, and pRSV-REV and a lentiviral vector coding for the genes of interest. Target cells were transduced with lentivirus supernatant in the presence of 4 μg/ml polybrene by spin infection (1000x*g*, 2h, 33°C). AKBM and Akata B cells, MJS, and 293T cells were transduced with human CD1d and selected using zeocin (200–500 μg/ml).

MJS cells were retrovirally transduced in the presence of 12μg/ml retronectin with the pLZRS-HCMV pp65-IRES-trNGFR vector to generate MJS-pp65 cells. Cells were FACS sorted for surface trNGFR expression. MJS or MJS-pp65 cells were transduced with eGFP-myc-tagged HLA-A2 or HLA-A2 to generate MJS-GFPmycA2 and MJS-A2/pp65 cells, respectively.

To generate CHO-A2-CD1d and Lec2-A2-CD1d cells, CHO and Lec2 cells were first transduced with human codon-optimized β_2_m and selected with zeocin (500 μg/ml). Subsequently, cells were transduced to express HLA-A2 and human CD1d. HLA-A2^+^CD1d^+^ cells were FACS sorted using a FACS Aria III (BD).

To study gp150 in MJS cell lines, gp150 or the variants HA-gp150 or HA-gp150ΔC were expressed from a lentiviral pCMV-IRES-GFP or pCMV-IRES-puro^R^ vector. The empty pCMV-IRES-GFP vector served as a control. MJS cell lines were transduced and analysed 3 to 5 days post transduction. Floating MJS cells were harvested and analysed 3–4 days post transduction. CHO and AkataΔgp150 cell lines were transduced, as indicated, with gp150, HA-gp150, or HA-gp150ΔC, which were expressed from the dual promotor lentiviral vector pEF1a-/pGK-GFP-T2A-puro^R^. CHO cells were analysed up to 10 days post transduction. AkataΔgp150 cells were selected using puromycin and analysed up to 4 weeks post transduction.

### Transient transfection

For transient transfections, 293T cells were seeded in 12w plates one day prior to transfection. Cells were transfected with 1 μg plasmid DNA coding for EBV proteins using Lipofectamine2000 (Invitrogen) according to the manufacturer’s protocol. Cells were analysed by flow cytometry two days post transfection.

### Flow cytometry, cell sorting, and cell viability assay

To assess cell surface levels of indicated molecules, cells were stained with antibodies of indicated specificity in PBS supplemented with 0,5% BSA and 0,02% sodium azide. For intracellular stains (ICS), cells were fixed in 2% PFA and permeabilized in ICS-FACS buffer (PBS, 2% FCS, 0,5% saponine). Subsequent staining and washing steps were performed in ICS-FACS buffer. All stainings were performed at 4°C. Cells were fixed prior to analysis in FACS buffer containing 2% PFA. Samples were subjected to flow cytometry using a LSR II or Canto II (BD Biosciences). Flow cytometry data were analysed using FlowJo (Tree star) software.

For cell sorting, cells were stained with indicated antibodies in PBS supplemented with 0,5% BSA for 1 hour on ice. Cells were washed in supplemented PBS and subjected to sorting by flow cytometry using an Aria III (BD Biosciences). Sorted cells were washed and prepared for use in T cell assays or Western blot analysis.

To determine cell viability by flow cytometry, unfixed cells were incubated with 7-AAD for 5 min at RT and subjected to flow cytometry. Live cells excluded the dye, whereas dead cells become 7-AAD positive.

### Functional assays

MJS-HLA-A2/pp65 cells were transduced with two different doses of lentivirus for gp150 expression or an empty vector control. Three days post-transduction, floating and adherent cells were harvested and adherent cells were FACS sorted for GFP^high^ and GFP^int^ cells. In 96w U-bottom plates, triplicates of 5000 cells were incubated with equal numbers of A2/pp65-specific CD8^+^ T cells or DR3/MiHa-specific CD4^+^ T cells overnight. Culture supernatants were collected to determine IFN-γ secretion by ELISA (Affymetrix) according to manufacturer’s protocol. Standard deviation was determined over biological triplicates.

For MHC/peptide-TCR interactions, 293K^b^ cells were transfected with gp150 or an empty vector control. One day post-transfection, cells were peptide-pulsed for 2h with either 1 μM ovalbumin-derived SIINFEKL peptide or the control peptide SSIEFARL. Cells were harvested and cell surface-stained using the 25D1.16 Ab, which is specific for K^b^/SIINFEKL complexes. Samples were subjected to flow cytometry. Mean fluorescence intensity was determined for triplicates in each experiment. Statistical analysis was performed on three independent experiments using Student’s t-test (two-tailed).

### Confocal microscopy

The localization of C-terminally GFP-tagged HLA-A2 expressed in MJS cells was determined by confocal microscopy. HA-gp150ΔC-transduced cells were harvested and cell surface stained for HLA-A2 (BB7.2) and gp150 (anti-HA) as performed for flow cytometry. Cell suspension was applied on a glass coverslip. Cells were analysed using a Leica TCS SP5 confocal microscope. Microscopy images were adjusted in levels in a linear fashion. For quantification, around 30 single cells were analysed using a composite colour image of the three different channels. Images of single-stained cells (BB7.2 or anti-HA) were used to define the amount of fluorescence leakage into the regarding other channel. Prior to image quantification of double-stained cells, measured pixel intensity was corrected for the determined leakage. Pixel intensity was determined for the outer 8 pixels of the selected objects. Statistical analysis was performed using an unpaired t-test (two-tailed).

### Western blot

Post-nuclear cell lysates were prepared using NP40 lysis buffer (50 mM Tris HCl (pH 7.5), 150 mM NaCl, 0.5% Igepal-CA630) containing protease inhibitors (Roche, Protease cocktail), as described previously [15]. For treatment with glycosidases, lysates were denatured at 95°C for 5 min prior to incubation with Endo H or PNGase F (both NEB) at 37°C for 1h. Proteins were separated by SDS-PAGE and blotted onto PVDF membranes. Membranes were blocked using 5% milk in PBS. The indicated primary antibodies were used to probe membranes overnight at 4°C in 1% milk in PBST (PBS, 0,1% Tween 20). Incubation with secondary antibody was performed for 2 hours at RT. Antibody-reactive bands were detected by ECL and signal was captured by film.

### Protease inhibitor treatment

Cells were cultured overnight in the presence of 25 μM chloroquine (provided by Dr. A de Wilde) or 15 nM epoxomicin (provided by Dr. H Overkleeft) to inhibit proteolysis by endolysosomal proteases or cytoplasmic proteasomes, respectively.

### Inhibition of sialylation

Optimal concentration of the fluorinated P-3F_ax_-Neu5Ac inhibitor was determined and effectiveness of treatment was monitored by binding of the biotinylated lectins SNAI (*Sambucus nigra*), MALII (*Maackia amurensis*), and PNA (*Ara chishypogaea*, all purchased by EY Laboratories). Lectin stains were performed in carbo-free blocking solution (Vector Laboratories) as described previously [42]. Cells were treated with 500 μM fluorinated P-3F_ax_-Neu5Ac inhibitor or with the control compound P-Neu5Ac 1 day prior to lentiviral transduction. Cells were cultured in presence of inhibitor for additional four days and analysed by flow cytometry.

### Neuraminidase treatment

Neuraminidase treatment was performed in FACS buffer containing 1U/μl neuraminidase (NEB), at 37°C for 45–60 min. Subsequently, cells were washed and stained in cold FACS buffer. Neuraminidase treatment was monitored by loss of binding of the biotinylated or FITC-conjugated lectin WGA (*Triticum vulgaris*). Stained cells were subjected to flow cytometry.

### Statistical analysis

Fluorescence intensity signals of each analysed cell were logarithmically transformed and 1000 cells per parameter (Figs 6 and 7B, gp150^-^/gp150^+^, Fig 7C, latent/lytic) were randomly picked. For each parameter, two “treatments” were compared (Figs 6D, CHO vs Lec2, 6H, control vs inhibitor, 6L and 7B, untreated vs neuraminidase, 7C, WT vs Δgp150). Whether treatment differences vary over gp150 expression has been explored by means of evaluating a statistical interaction between these factors in an analysis of variance model. Statistical analysis was performed using two-way ANOVAs and significance of the interaction terms (ctrl/gp150*untreated/treated) was assessed using SPSS (version 20.0.0.2). p-values < 0.05 were considered statistically significant.

## Supporting Information

S1 Fig A) EBV^+^ AKBM-CD1d BL cells were treated for indicated periods with anti-human IgG Ab to induce viral replication. EBV-producing cells were identified by induced expression of the lytic cycle reporter rat CD2-GFP. Surface levels of the Ag-presenting molecules HLA I, II, and CD1d were determined by flow cytometry. Histograms depict overlays to allow comparison of latently (rat CD2-GFP^-^) and lytically (rat CD2-GFP^+^) infected B cells. B) 293T-CD1d cells were transfected with expression vectors encoding late EBV glycoproteins. Glycoproteins known to require a viral interaction partner were transfected together (BMRF2/BDLF2 and gM/gN). EBV protein expression was deduced from coexpression of GFP (BMRF2/BDLF2) or on the basis of a C-terminal tag (gM/gN, gB, gp350, gp150). Cell surface HLA I or TfR was stained prior to an intracellular staining for the tagged EBV proteins. Surface levels were compared between non-transfected and transfected cells.
(TIF)

S2 Fig MJS-CD1d cells were transduced with lentiviruses encoding (A) the indicated EBV immune evasion gene products (BNLF2a, BILF1, gp42+gH+gL) or only IRES-GFP (vector) and (B,C) gp150-IRES-GFP. Surface levels of HLA I, HLA II, and CD1d (A) as well as TfR (B) or CD10 and CD54 (C) were determined by flow cytometry on non-permeabilized cells. Histograms depict a comparison of GFP^-^ control (non-transduced) and GFP^+^ EBV protein-expressing (transduced) cells. C) A dose range of pCMV-gp150-IRES-GFP lentivirus was used for transduction. Total gp150 expression levels in permeabilized cells were determined by intracellular staining with an Ab specific for gp150’s cytoplasmic tail.
(TIF)

S3 Fig A) The adherent MJS cell line was transduced either with the gp150-IRES-GFP lentivirus or an IRES-GFP control. Three days post transduction, both the floating and adherent fractions of transduced cells were subjected to flow cytometry. The proportion of floating cells was larger for gp150-transduced cells than for control cells and, additionally, the gp150^+^ floating cells were enriched for gp150 expression (reflected by higher GFP levels compared to the adherent cells). These observations suggested that high levels of gp150 expression induce loss of cell adherence. To exclude that the higher gp150 levels were cytotoxic, the viability of floating and adherent fractions of transduced cells from the same culture dish was determined by incubation with the live exclusion dye 7-aminoactinomycin D (7AAD) followed by flow cytometry analysis. As controls served the adherent fraction of untreated MJS-CD1d cells (control) and the adherent and floating fractions of cells treated with toxic concentrations of the proteasome inhibitor epoxomicin (epox; 200 nM) for 24h. In the FSCxSSC dot plots, the live gates are depicted for the cell populations analyzed for GFP levels (transduction efficiencies) and 7AAD exclusion (viability). An additional gate on population 1 in the floating epoxomicin-treated cells shows that the 7AAD was effective in penetrating dead cells. Among both the adherent and the floating cells transduced with either control or gp150 lentivirus, only very few 7AAD^+^ (dead) cells were present, indicating that gp150 does not cause gross cytotoxicity. B) The floating cell fraction from the experiment depicted in Fig 2A was analyzed by flow cytometry, as described in the legend to Fig 2A.
(TIF)

S4 Fig Three days after lentiviral transduction, four different populations were FACS sorted from MJS-CD1d cells that were transduced with gp150-IRES-GFP or control IRES-GFP viruses: GFP- (1, non-transduced) and GFP+ (2, vector) cells were isolated from control cells and the GFP+ cells from the gp150-transduction were further separated into gp150low (3, HLA Ihigh) cells and gp150high (4, HLA Ilow) cells on the basis of the extent of HLA I downregulation. The sorted cell populations were lysed for analysis by immunoblot (see Fig 4B).
(TIF)

S5 Fig Intact MJS-CD1d-gp150 and control-IRES-GFP cells were treated with decreasing amounts of neuraminidase (5–0,008U/ml) for 60 min at 37°C and compared to untreated cells. Effectiveness of neuraminidase treatment was monitored by WGA-FITC binding. CD1d surface levels were determined by flow cytometry. (TIF)

S6 Fig A) EBV^+^ AKBM, Akata wt and Δgp150 BL cells were treated with anti-human IgG to induce the viral lytic cycle. Twenty hours later, expression of several EBV proteins—BZLF1 (immediate-early), BGLF5 (early), and gp150 and surface gp350 (both late)—was determined using flow cytometry. B) Akata wt and Δgp150 BL cells were treated with anti-human IgG for 20 hours. Surface expression of the cellular proteins HLA I, CD1d, and CD86 was determined using flow cytometry as described in Fig 7C.
(TIF)
