# Supplementary figures and images for: The Epstein-Barr Virus Glycoprotein gp150 Forms an Immune-Evasive Glycan Shield at the Surface of Infected Cells
Source: PLoS Pathog. 2016 Apr 14;12(4):e1005550. doi: 10.1371/journal.ppat.1005550 (PMC4831753; doi:10.1371/journal.ppat.1005550)

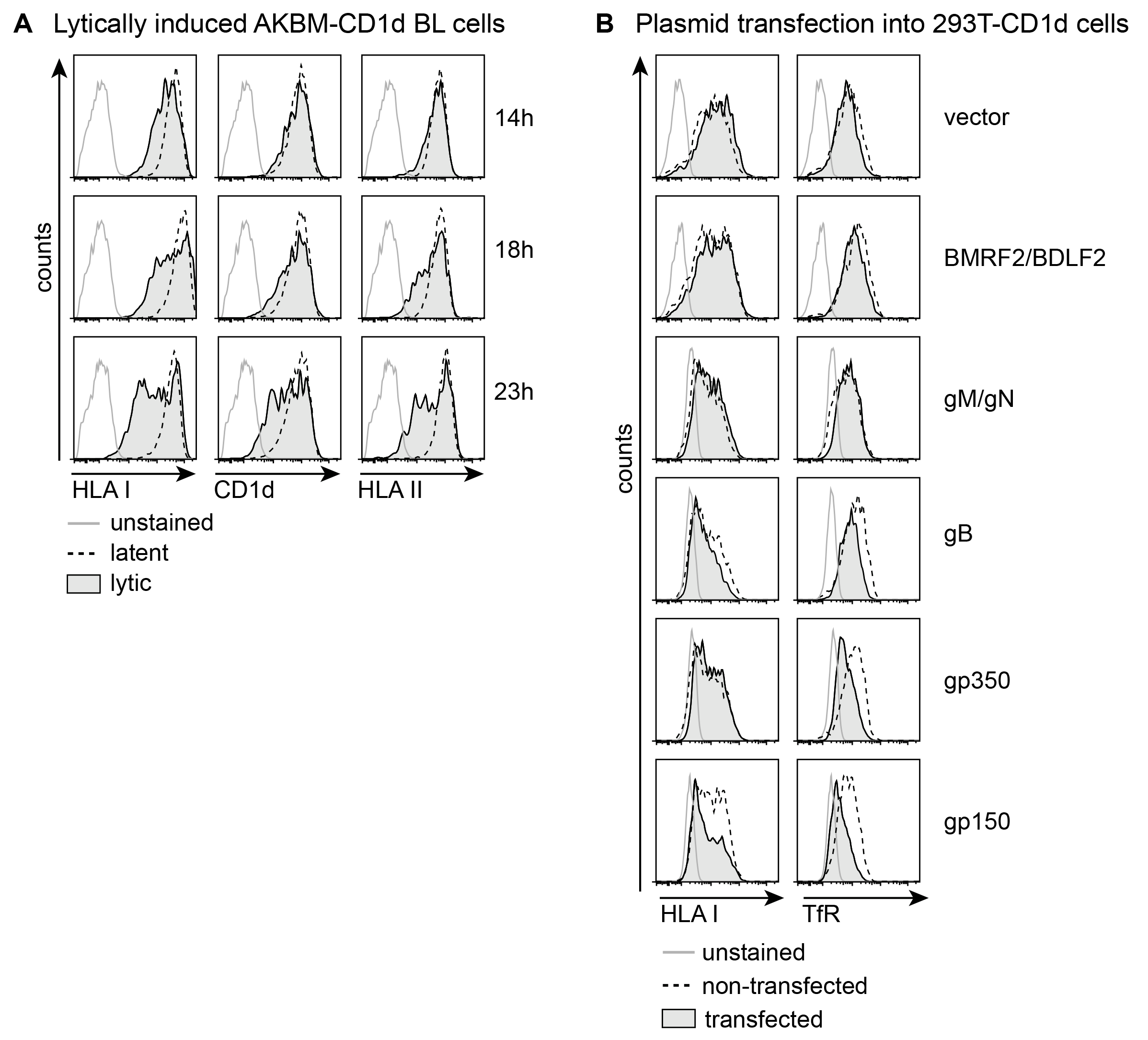

Supplement: S1 Fig — A) EBV+ AKBM-CD1d BL cells were treated for indicated periods with anti-human IgG Ab to induce viral replication. EBV-producing cells were identified by induced expression of the lytic cycle reporter rat CD2-GFP. Surface levels of the Ag-presenting molecules HLA I, II, and CD1d were determined by flow cytometry. Histograms depict overlays to allow comparison of latently (rat CD2-GFP-) and lytically (rat CD2-GFP+) infected B cells. B) 293T-CD1d cells were transfected with expression vectors encoding late EBV glycoproteins. Glycoproteins known to require a viral interaction partner were transfected together (BMRF2/BDLF2 and gM/gN). EBV protein expression was deduced from coexpression of GFP (BMRF2/BDLF2) or on the basis of a C-terminal tag (gM/gN, gB, gp350, gp150). Cell surface HLA I or TfR was stained prior to an intracellular staining for the tagged EBV proteins. Surface levels were compared between non-transfected and transfected cells. (TIF) [file ppat.1005550.s001.tif]

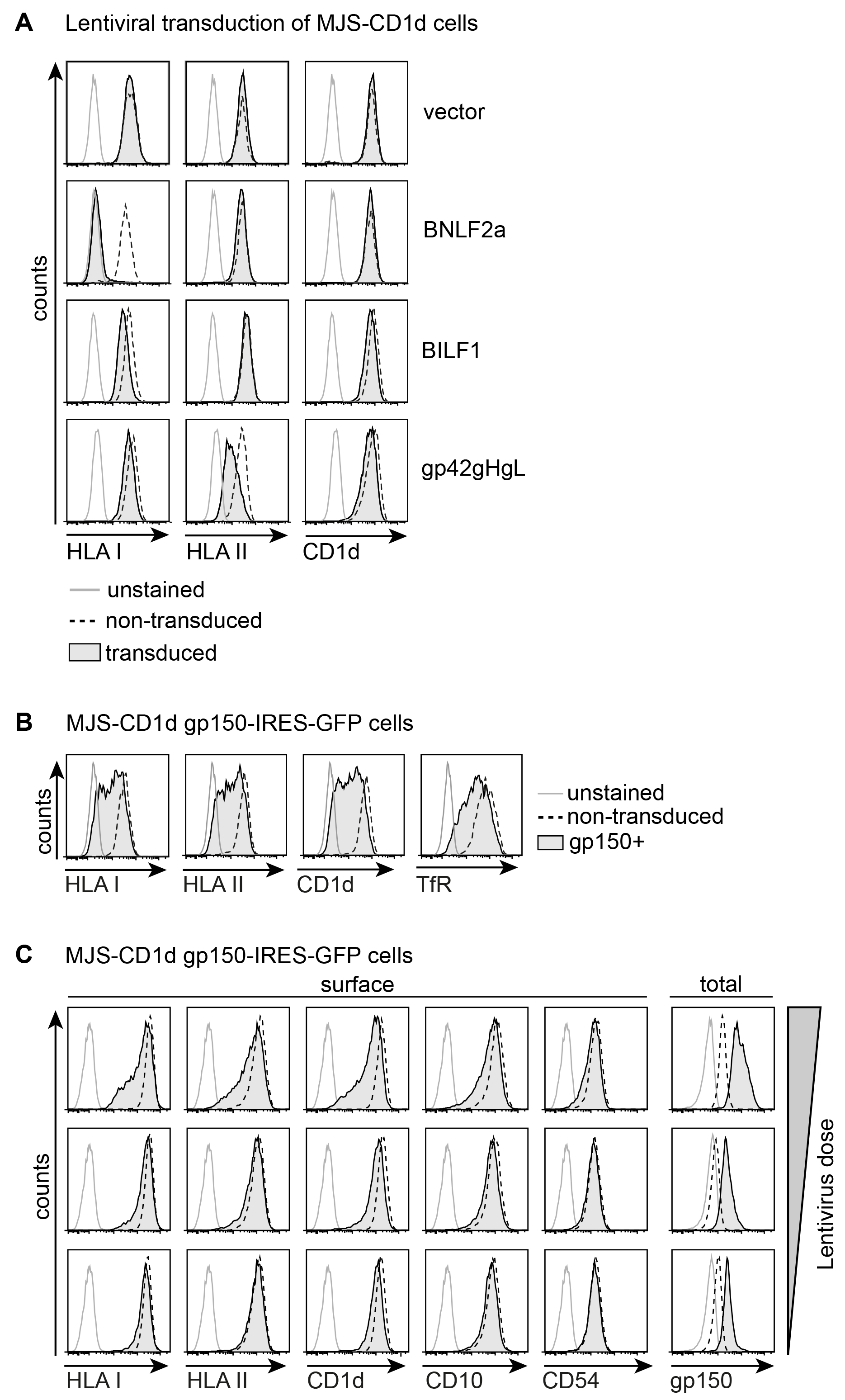

Supplement: S2 Fig — MJS-CD1d cells were transduced with lentiviruses encoding (A) the indicated EBV immune evasion gene products (BNLF2a, BILF1, gp42+gH+gL) or only IRES-GFP (vector) and (B,C) gp150-IRES-GFP. Surface levels of HLA I, HLA II, and CD1d (A) as well as TfR (B) or CD10 and CD54 (C) were determined by flow cytometry on non-permeabilized cells. Histograms depict a comparison of GFP- control (non-transduced) and GFP+ EBV protein-expressing (transduced) cells. C) A dose range of pCMV-gp150-IRES-GFP lentivirus was used for transduction. Total gp150 expression levels in permeabilized cells were determined by intracellular staining with an Ab specific for gp150’s cytoplasmic tail. (TIF) [file ppat.1005550.s002.tif]

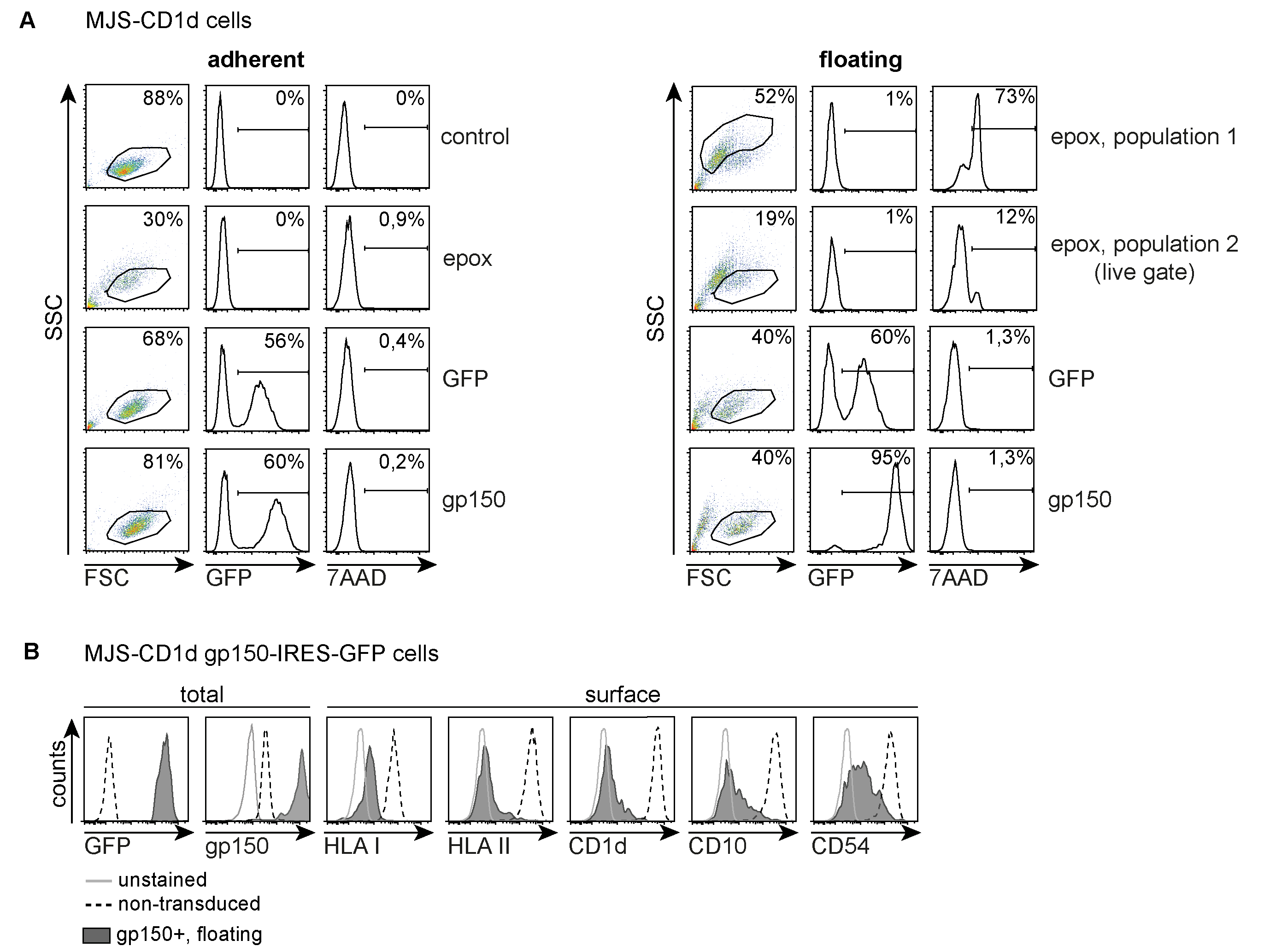

Supplement: S3 Fig — A) The adherent MJS cell line was transduced either with the gp150-IRES-GFP lentivirus or an IRES-GFP control. Three days post transduction, both the floating and adherent fractions of transduced cells were subjected to flow cytometry. The proportion of floating cells was larger for gp150-transduced cells than for control cells and, additionally, the gp150+ floating cells were enriched for gp150 expression (reflected by higher GFP levels compared to the adherent cells). These observations suggested that high levels of gp150 expression induce loss of cell adherence. To exclude that the higher gp150 levels were cytotoxic, the viability of floating and adherent fractions of transduced cells from the same culture dish was determined by incubation with the live exclusion dye 7-aminoactinomycin D (7AAD) followed by flow cytometry analysis. As controls served the adherent fraction of untreated MJS-CD1d cells (control) and the adherent and floating fractions of cells treated with toxic concentrations of the proteasome inhibitor epoxomicin (epox; 200 nM) for 24h. In the FSCxSSC dot plots, the live gates are depicted for the cell populations analyzed for GFP levels (transduction efficiencies) and 7AAD exclusion (viability). An additional gate on population 1 in the floating epoxomicin-treated cells shows that the 7AAD was effective in penetrating dead cells. Among both the adherent and the floating cells transduced with either control or gp150 lentivirus, only very few 7AAD+ (dead) cells were present, indicating that gp150 does not cause gross cytotoxicity. B) The floating cell fraction from the experiment depicted in Fig 2A was analyzed by flow cytometry, as described in the legend to Fig 2A. (TIF) [file ppat.1005550.s003.tif]

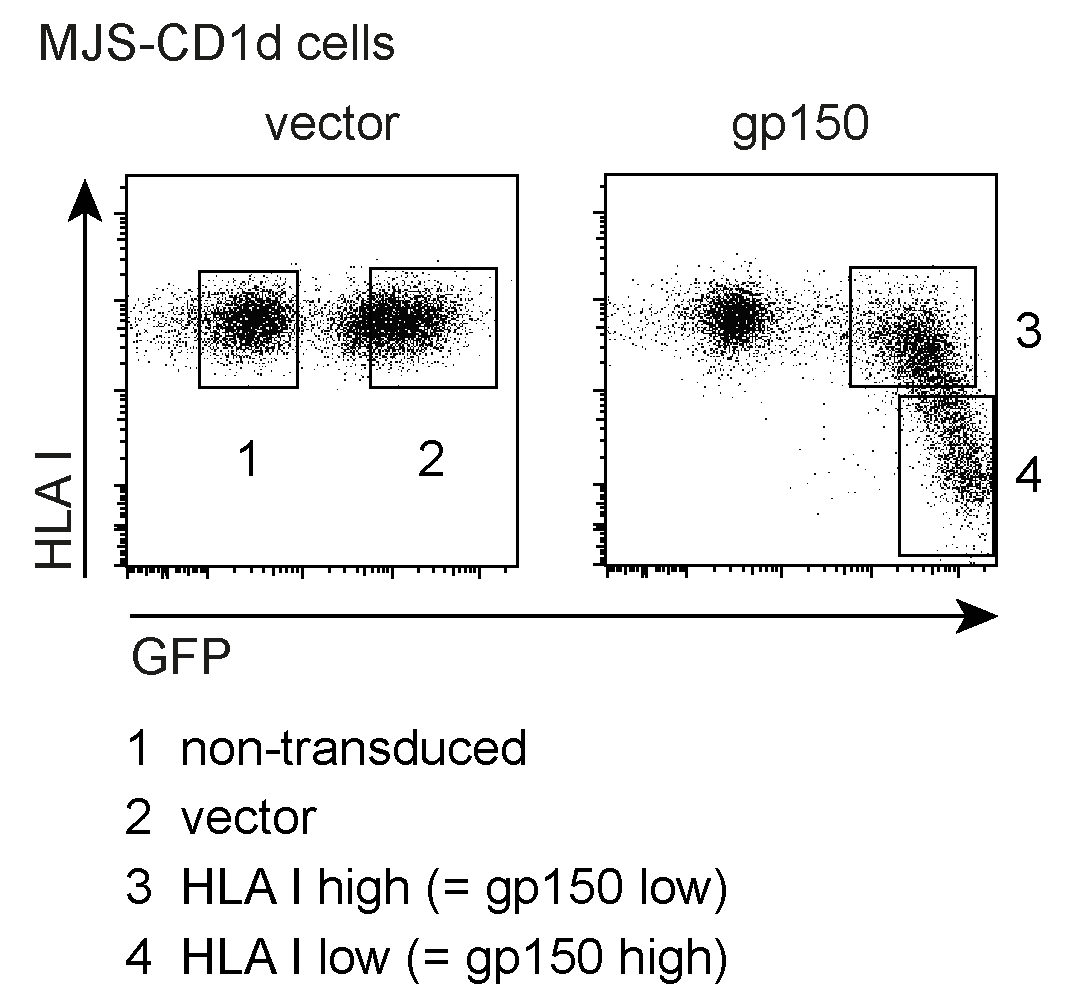

Supplement: S4 Fig — The sorted cell populations were lysed for analysis by immunoblot (see Fig 4B). (TIF) [file ppat.1005550.s004.tif]

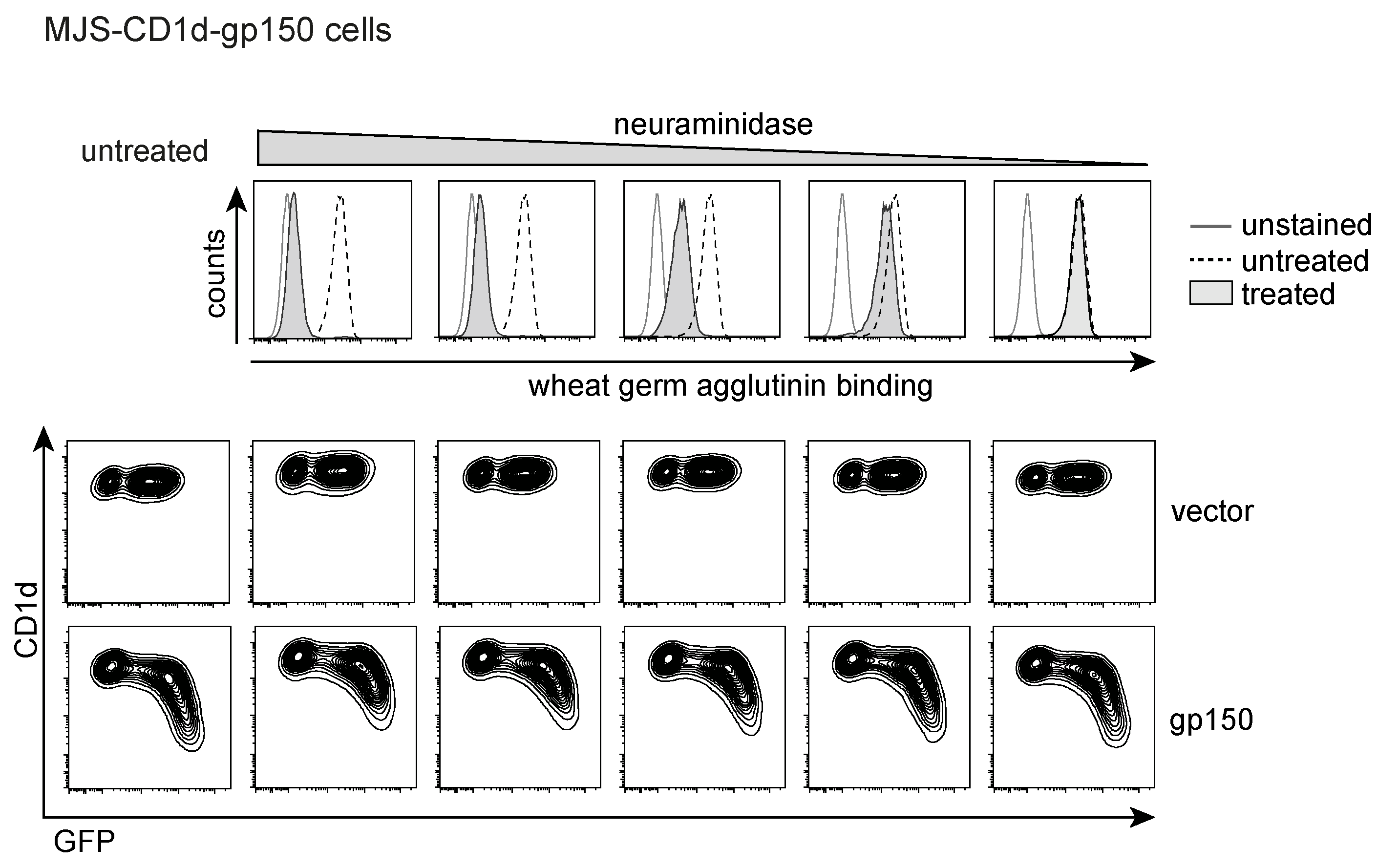

Supplement: S5 Fig — (TIF) [file ppat.1005550.s005.tif]

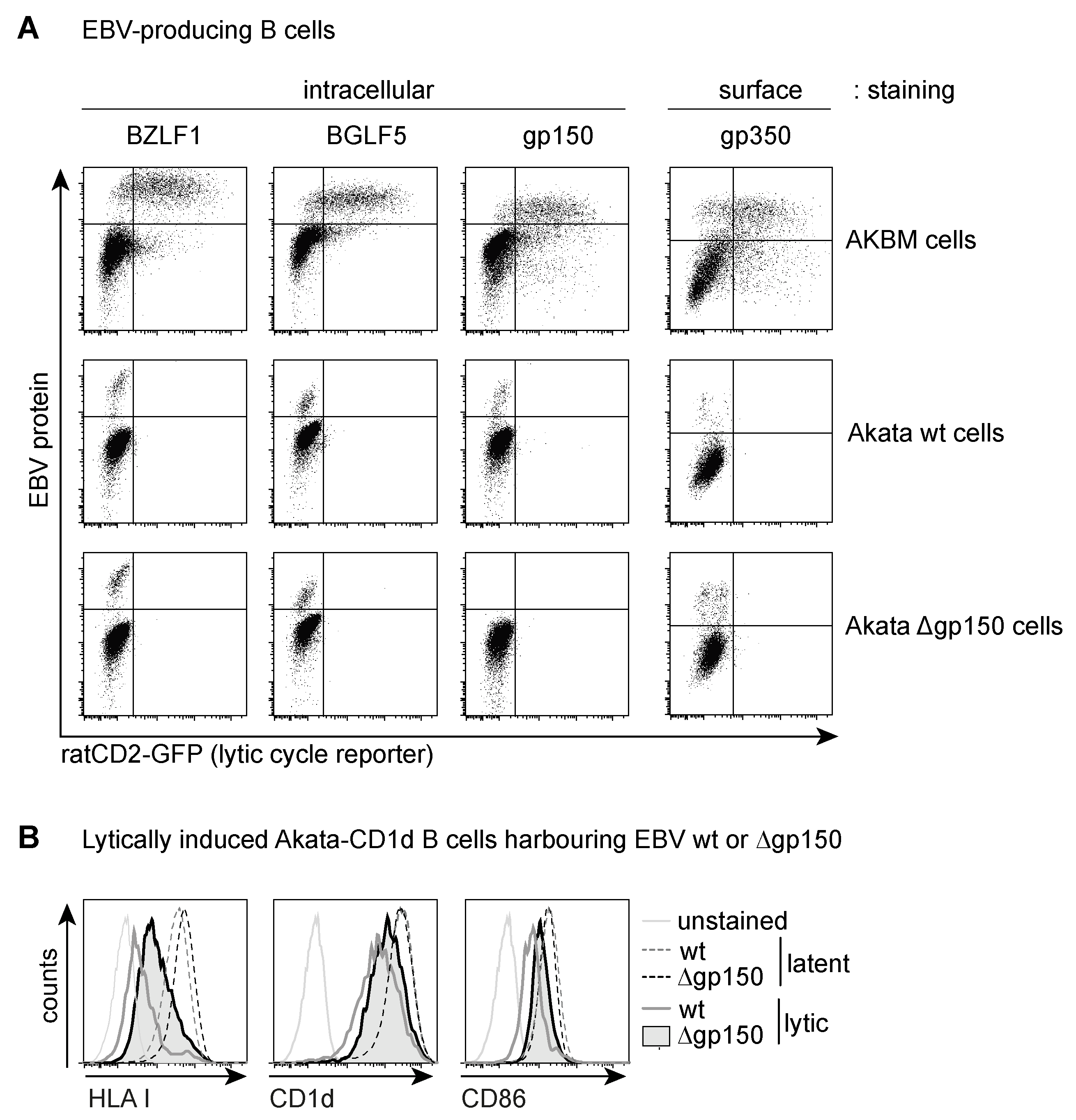

Supplement: S6 Fig — A) EBV+ AKBM, Akata wt and Δgp150 BL cells were treated with anti-human IgG to induce the viral lytic cycle. Twenty hours later, expression of several EBV proteins—BZLF1 (immediate-early), BGLF5 (early), and gp150 and surface gp350 (both late)—was determined using flow cytometry. B) Akata wt and Δgp150 BL cells were treated with anti-human IgG for 20 hours. Surface expression of the cellular proteins HLA I, CD1d, and CD86 was determined using flow cytometry as described in Fig 7C. (TIF) [file ppat.1005550.s006.tif]
